# Tanzawaic acids I–L: Four new polyketides from *Penicillium* sp. IBWF104-06

**DOI:** 10.3762/bjoc.10.20

**Published:** 2014-01-22

**Authors:** Louis P Sandjo, Eckhard Thines, Till Opatz, Anja Schüffler

**Affiliations:** 1 Johannes-Gutenberg-University Mainz, Institute of Organic Chemistry, Duesbergweg 10–14, D-55128 Mainz, Germany; 2 Institute of Biotechnology and Drug Research, Erwin-Schroedinger-Strasse 56, D-67663, Kaiserslautern, Germany; 3 Johannes-Gutenberg-University Mainz, Institute of Biotechnology and Drug Research, Duesbergweg 10–14, D-55128 Mainz, Germany

**Keywords:** arohynapene, natural products, polyketides, structure elucidation, tanzawaic acid

## Abstract

Four new polyketides have been identified in culture filtrates of the fungal strain *Penicillium* sp. IBWF104-06 isolated from a soil sample. They are structurally based on the same *trans*-decalinpentanoic acid skeleton as tanzawaic acids A–H. One of the new compounds was found to inhibit the conidial germination in the rice blast fungus *Magnaporthe oryzae* at concentrations of 25 μg/mL.

## Introduction

Nature still represents the richest source of new antimicrobials which can be attractive as lead structures or biochemical tools for target identification for medical applications as well as for crop science. In both areas, the omnipresent development of resistance results in a demand for new active principles for which the structural diversity of nature holds an excellent supply. While natural products, their synthesis, evolution, and ecological role are of academic interest, compounds identified might also be relevant for application. In the course of investigations into the secondary metabolism of fungal species for the identification of novel lead structures for agrochemical and pharmaceutical research, we isolated a fungal strain from a soil sample. The culture filtrate extract of this organism was found to inhibit the conidial germination in the rice blast fungus *Magnaporthe oryzae*, one of the most devastating plant diseases on cultivated rice. Bioactivity-guided fractionation of organic extracts led to the isolation of a series of eight pure compounds, the structures of which were elucidated by 2D NMR spectroscopy as well as by mass spectrometry.

## Results and Discussion

**Figure 1:**
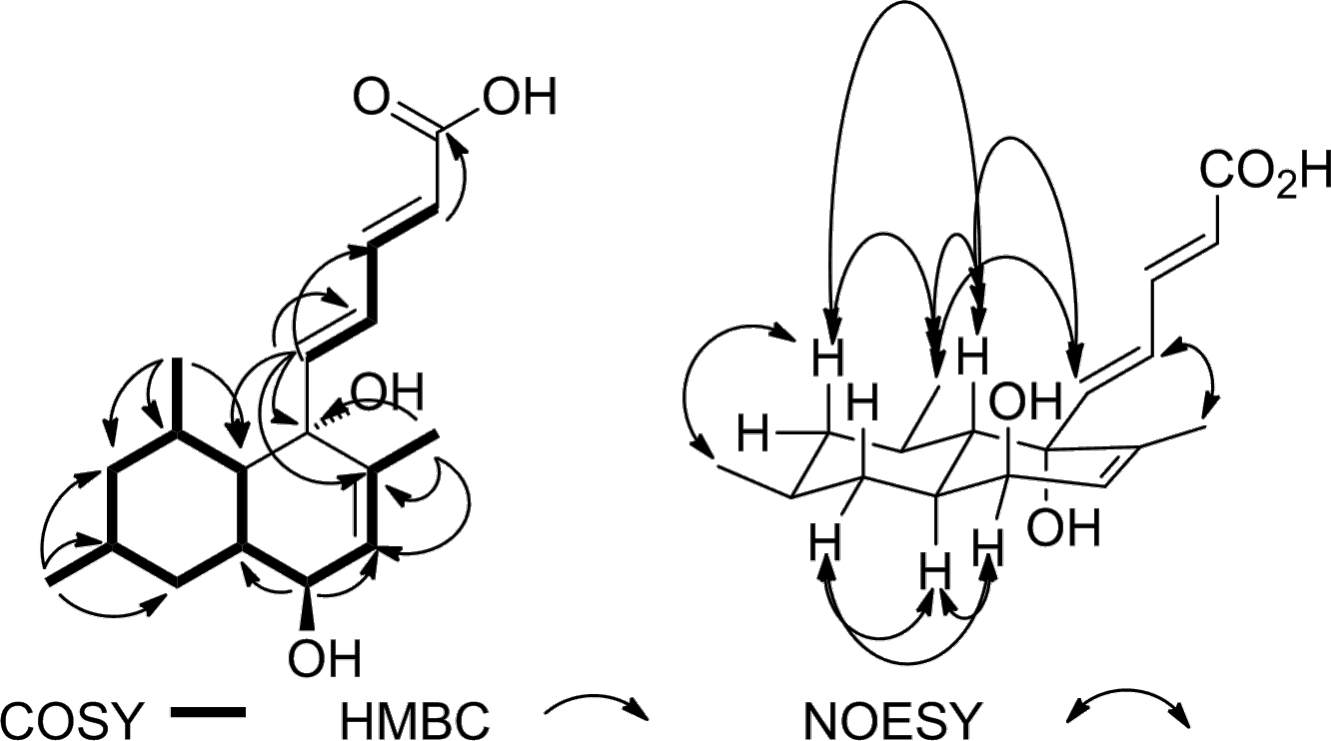
HMBC, COSY and NOESY correlations of compound **1**.

**Structure elucidation.** Compound **1** has the molecular formula C_18_H_26_O_4_ determined on the basis of NMR and HRMS-ESI data which gave a pseudo-molecular ion at *m*/*z* 329.1744 (calcd for [M + Na]^+^: 329.1729). This composition accounted for six double bond equivalents. The NMR spectra of **1** (Table 1 and Table 2) displayed signals of a (2*E*,4*E*)-penta-2,4-dienoic acid moiety including a carbon of a conjugated carbonyl group at δ_C_ 170.9 and four olefinic resonances at δ_H_ 5.86 (d, *J* = 15.3 Hz)/δ_C_ 122.0, δ_H_ 7.28 (dd, *J* = 11.2, 15.3 Hz)/δ_C_ 145.9, δ_H_ 6.45 (dd, *J* = 11.2, 15.2 Hz)/128.5, and δ_H_ 6.10 (d, *J* = 15.2 Hz)/δ_C_ 144.8. Moreover, these spectra showed signals of three CH_3_ groups, two CH_2_ groups, six CH groups, and two quaternary carbons (Table 1 and Table 2). These data suggested a structural similarity of **1** to the tanzawaic acids, secondary metabolites from *Penicillium citrinum* [1]. COSY and HMBC correlations (Figure 1) revealed the presence of a *trans*-decalin scaffold containing six CH groups, two CH_2_ groups and two quaternary carbons as the hydrogen at δ_H_ 1.39 (H-7) showed correlations with hydrogens at δ_H_ 1.59 (H-12) and 0.72 (H-9) while the latter correlated with the proton at δ_H_ 1.45 (H-10). Likewise, the resonance at δ_H_ 1.45 had a correlation with the proton at δ_H_ 1.17 (H-11_ax_) which in turn correlated with those at δ_H_ 1.54 (H-11_eq_) and 1.59 (H-12) forming the western part of the bicyclic framework; further homonuclear interactions were revealed from H-12 (δ_H_ 1.59) to H-13 (δ_H_ 3.80) and H-14 (δ_H_ 5.69). The eastern part of the decalin system was established based on HMBC correlations between the olefinic proton at δ_H_ 5.69 (H-14) and the carbons C-12 (δ_C_ 41.4), C-13 (δ_C_ 67.6), and C-6 (δ_C_ 79.0) while the hydrogen of the oxymethine at δ_H_ 3.80 (H-13) correlated with the carbons C-7 (δ_C_ 49.8), C-14 (δ_C_ 127.0) and C-15 (δ_C_ 142.9). Based on the COSY and HMBC interactions as well as their 1D multiplicity, the CH_3_ groups at δ_H_ 0.90 (H-17), 1.09 (H-18) and 1.62 (H-16) were attached to the carbons resonating at δ_C_ 32.9 (C-10), 35.3 (C-8), and 142.9 (C-15) respectively, while the penta-2,4-dienoic acid unit was connected to the carbon at δ_C_ 79.0 (C-6).

**Table 1:** ^1^H NMR data of compounds **1**–**4** (CD_3_OD, 600 MHz).

Position	Compound **1**	Compound **2**	Compound **3**	Compound **4**

1	–	–	–	–
2	5.86 (1H, d, 15.3)	5.83 (1H, d, 15.3)	5.80 (1H, d, 15.3)	5.87 (1H, d, 15.1)
3	7.28 (1H, dd, 11.2, 15.3)	7.28 (1H, dd, 11.2, 15.3)	7.32 (1H, dd, 10.9, 15.3)	7.37 (1H, dd, 11.1, 15.1)
4	6.45 (1H, dd, 11.2, 15.2)	6.45 (1H, dd, 11.2, 15.5)	6.20 (1H, dd, 11.0, 15.0)	6.33 (1H, dd, 11.1, 15.6)
5	6.10 (1H, d, 15.2)	6.16 (1H, d, 15.5)	6.31 (1H, dd, 10.6, 15.0)	6.74 (1H, d,15.6)
6	–	–	2.40 (1H, t, 10.6)	–
7	1.39 (1H, m)	1.52 (1H, d, 11.0)	1.09 (1H, m)	1.56 (1H, m)
8	1.39 (1H, m)	1.75 (1H, m)	1.39 (1H, m)	2.03 (1H, m)
9	0.72 (1H_ax_, q, 11.8) 1.54 (1H_eq_, m)	0.78 (1H_ax_, q, 12.3) 1.57 (1H_eq_, m)	0.77 (1H_ax_, m) 1.65 (1H_eq_, *pseudo*-dd, 3.1, 13.3)	1.06 (1H_ax_, dd, 11.9, 13.9) 1.73 (1H_eq_, m)
10	1.45 (1H, d, 6.6)	1.50 (1H, m)	1.54 (1H, m)	–
11	1.17 (1H_ax_, q, 12.3) 1.54 (1H_eq_, m)	1.17 (1H_ax_, q, 12.4) 1.56 (1H_eq_, m)	0.72 (1H_ax_, m) 1.71 (1H_eq_, *pseudo*-dd, 3.0, 13.0)	1.33 (1H_ax_, t, 13.3) 1.69 (1H_eq_, m)
12	1.59 (1H, m)	1.61 (1H, m)	1.94 (1H, *pseudo*-t, 11.0)	2.36 (1H, m)
13	3.80 (1H, t*,* 4.4)	3.75 (1H, dd, 2.7, 5.8)	5.41 (1H, d, 10.0)	5.74 (1H, dd, 2.2, 9.2)
14	5.69 (1H, d, 5.2)	5.79 (1H, d, 6.1)	5.48 (1H, dd, 2.4, 10.0)	5.97 (1H, dd, 2.8, 9.2)
15	–	–	–	–
16	1.62 (3H, s)	1.63 (3H, s)	1.18 (3H, s)	1.89 (3H, s)
17	0.90 (3H, d, 6.4)	0.91 (3H, d, 6.4)	0.89 (3H, d, 6.5)	1.20 (3H, s)
18	1.09 (3H, d, 5.3)	0.91 (3H, d, 6.4)	0.98 (3H, d, 6.3)	0.98 (3H, d, 6.2)

**Table 2:** ^13^C NMR data of compounds **1**–**4** (CD_3_OD, 150 MHz).

Position	Compound **1**	Compound **2**	Compound **3**	Compound **4**

1	170.9	170.9	170.9	170.9
2	122.0	121.3	120.6	120.8
3	145.9	146.2	146.7	147.1
4	128.5	127.8	130.5	131.0
5	144.8	153.7	147.5	142.3
6	79.0	76.1	58.8	133.4
7	49.8	49.1	51.1	49.9
8	35.3	34.3	37.2	29.1
9	47.1	47.1	48.1	49.3
10	32.9	33.0	33.7	70.1
11	39.1	39.0	42.8	44.0
12	41.4	39.9	44.7	35.9
13	67.6	68.0	132.9	137.3
14	127.0	128.0	135.2	131.0
15	142.9	140.9	74.5	133.8
16	18.1	19.1	24.6	19.8
17	23.1	23.1	22.8	31.3
18	23.0	24.2	23.8	23.4

The relative configuration of the stereogenic centers was assigned based on NOESY data (Figure 1) and the previous work reported by Malmstrøm et al. on the stereochemistry of two related compounds, tanzawaic acids E and F [2].

The NOESY spectrum showed contacts (Figure 1) between the proton of the oxymethine group at δ_H_ 3.80 (H-13) and the protons at δ_H_ 1.54 (H-11_eq_) and 1.59 (H-12) suggesting an axial orientation of the OH group at C-13. The proton at δ_H_ 1.17 (H-11_ax_) correlated with those at δ_H_ 0.90 (H-17), 0.72 (H-9_ax_), and 1.39 (H-8) while H-9_ax_ showed same interactions with the methyl protons at δ_H_ 1.09 (H-18) and 0.90 (H-17) suggesting them to be oriented equatorially. The same orientation was suggested for the unsaturated side chain since correlations were found between the proton at δ_H_ 6.10 (H-5) and those at 1.09 (H-18) and 1.39 (H-7).

The foregoing data in conjunction with those of the structurally related tanzawaic acid E [2] led to the identification of **1** as a new member of this compound class and it was given the name tanzawaic acid I (Figure 5).

**Figure 2:**
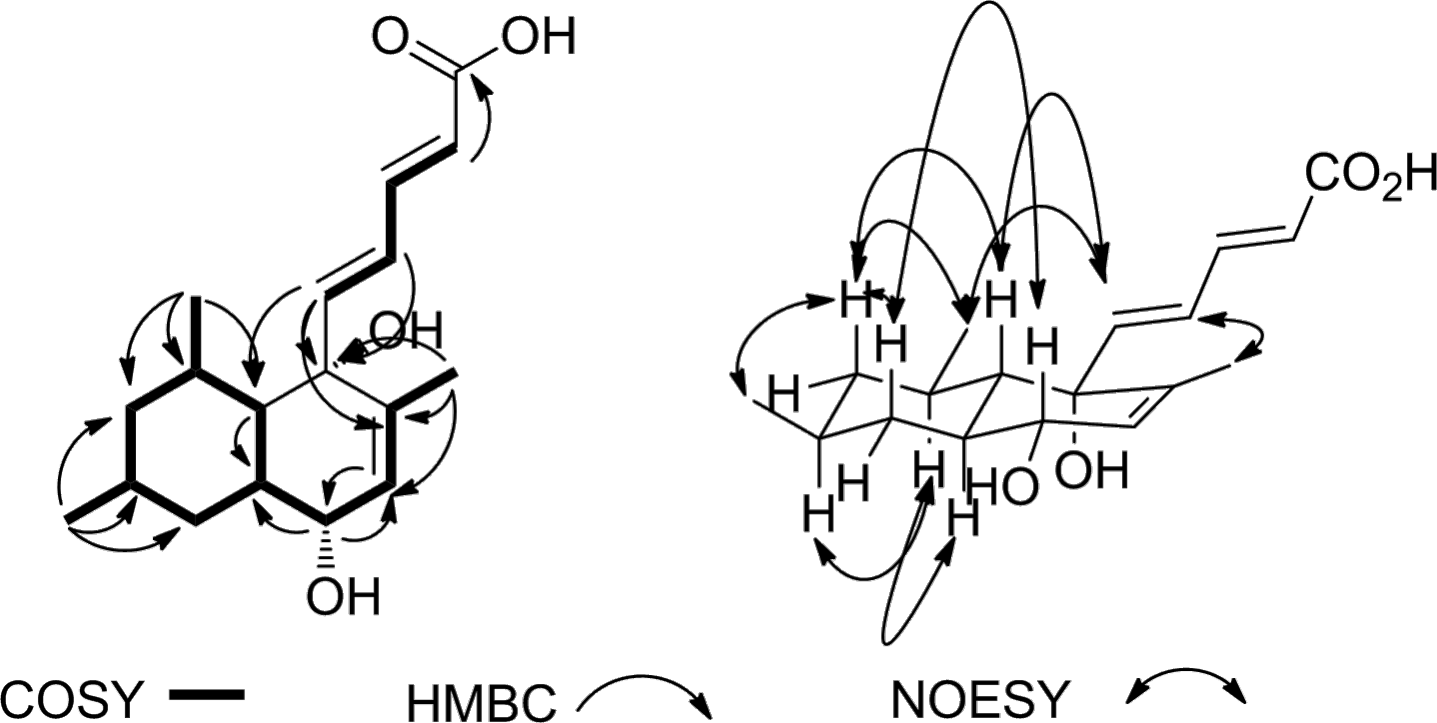
HMBC, COSY and NOESY correlations of compound **2**.

Compound **2** has the molecular formula C_18_H_26_O_4_ deduced from its NMR and HRMS-ESI data which revealed a pseudo-molecular ion at *m*/*z* 329.1736 (calcd for [M + Na]^+^: 329.1729). As in compound **1**, this composition accounted for six double bond equivalents. The NMR spectra of compound **2** exhibited features similar to those of **1**. Signals of a (2*E*,4*E*)-penta-2,4-dienoic acid moiety (δ_H_ 5.83 (d, *J* = 15.3 Hz, H-2)/δ_C_ 121.3, δ_H_ 7.28 (dd, *J* = 11.2, 15.3 Hz, H-3)/δ_C_ 146.2, δ_H_ 6.45 (dd, 11.2, 15.5 Hz, H-4)/δ_C_ 127.8, and δ_H_ 6.16 (d, *J* = 15.5 Hz, H-5)/δ_C_ 153.7 and δ_C_ 170.9 (C-1)) and a *trans*-decalin bearing three methyl groups (δ_H_ 0.91 (d, *J* = 6.4 Hz, H-17)/δ_C_ 23.1, 0.91 (d, *J* = 6.4 Hz, H-18)/δ_C_ 24.2 and δ_H_ 1.63 (s, H-16)/δ_C_ 19.1) were found as well as a trisubstituted double bond (δ_H_ 5.79 (d, *J* = 6.1 Hz, H-14)/δ_C_ 128.0 and δ_C_ 140.9 (C-15)), an oxymethine (δ_H_ 3.75 (dd, *J* = 2.7, 5.8 Hz, H-13)/δ_C_ 68.0), and a tertiary alcohol (δ_C_ 76.1, C-6). A careful inspection of COSY and HMBC data (Figure 2) of compound **2** suggested a diastereomeric relationship with **1** which was substantiated by the different optical rotations of –262 and +73.7 for compounds **1** and **2**, respectively.

In compound **2**, NOESY correlations (Figure 2) observed between H-11_ax_ (δ_H_ 1.17) and H-9_ax_ (δ_H_ 0.78), H-13 (δ_H_ 3.75) and H-7 (δ_H_ 1.52) revealed the oxymethine at δ_H_ 3.75 (dd, *J* = 2.7, 6.0 Hz)/δ_C_ 68.0 to be responsible for the diastereomery between **1** and **2** due to inversion of the configuration at C-13. Further NOESY correlations were observed between the proton resonating at δ_H_ 0.78 (H-9_ax_) and the methyl protons at δ_H_ 0.91 (Me-17 and Me-18) while H-18 showed the same interactions with the olefinic protons at δ_H_ 6.16 (H-5) and 6.45 (H-4). This observation suggested **2** to have the same relative configuration as **1** at C-6, C-7, C-8, and C-10. The above-mentioned data allowed to identify compound **2** as another member of the tanzawaic acid family and was given the name tanzawaic acid J.

**Figure 3:**
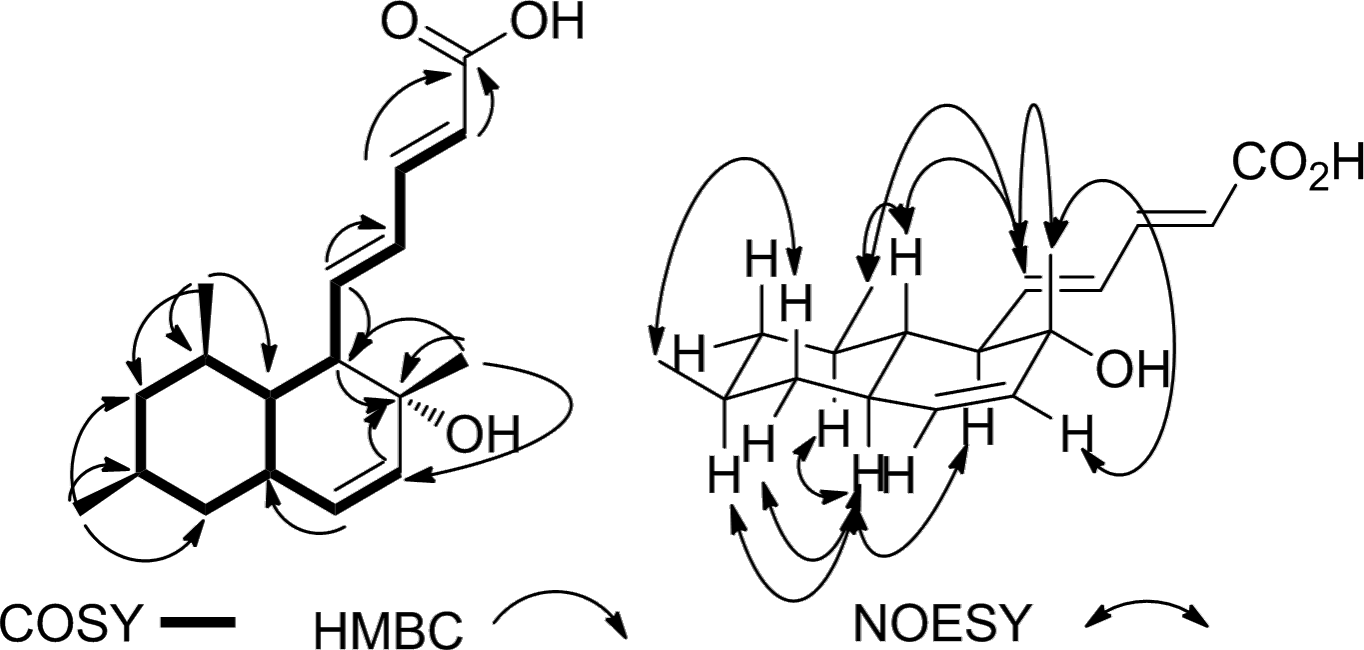
HMBC, COSY and NOESY correlations of compound **3**.

The molecular formula of compound **3**, C_18_H_26_O_3_ was established by NMR in conjunction with HRMS-ESI which gave a pseudo-molecular ion at *m*/*z* 313.1788 (calcd for [M + Na]^+^: 313.1780). This composition required six double bond equivalents and differed from those of compounds **1** and **2** by one oxygen atom. This observation was supported by the absence of oxymethine signals in the NMR spectra. However, features similar to those of the (2*E*,4*E*)-penta-2,4-dienoic acid moiety of compounds **1** and **2** and some signal patterns of the *trans*-decalin part different from those of the two other compounds were observed. The resonance of H-5 (δ_H_ 6.31, *J* = 10.6, 15.0 Hz) was found as a doublet of doublets suggesting the side chain to be attached to a methine carbon in the bicyclic portion. This conclusion was confirmed by a COSY-correlation to H-6 [δ_H_ 2.40 (t, *J* = 10.6 Hz)]. Furthermore, two signals of a *cis* olefin were unveiled at δ_H_ 5.41 (d, *J* = 10.0 Hz)/δ_C_ 132.9 and δ_H_ 5.48 (dd, *J* = 2.4, 10.0 Hz)/δ_C_ 135.2 and originated from the protons bound to C-13 and C-14. This supposition was consistent with diagnostic HMBC correlations connecting the protons [δ_H_ 1.18 (s)] of the C-16 methyl group to C-6 (δ_C_ 58.8), C-15 (δ_C_ 74.5) as well as C-14 (δ_C_ 135.2) (Figure 3).

The NOESY spectrum of compound **3** revealed similar correlations as in its analogues **1** and **2** between the olefinic proton H-5 (δ_H_ 6.31) and the CH_3_ group attached to C-8 suggesting the same relative orientation of the side chain and the CH_3_ group (Figure 3) which is supported by a correlation between H-5 and the CH_3_ (C-16) group (δ_H_ 1.18). The complete assignment led to structure **3** as shown in Figure 5. The compound was named tanzawaic acid K.

**Figure 4:**
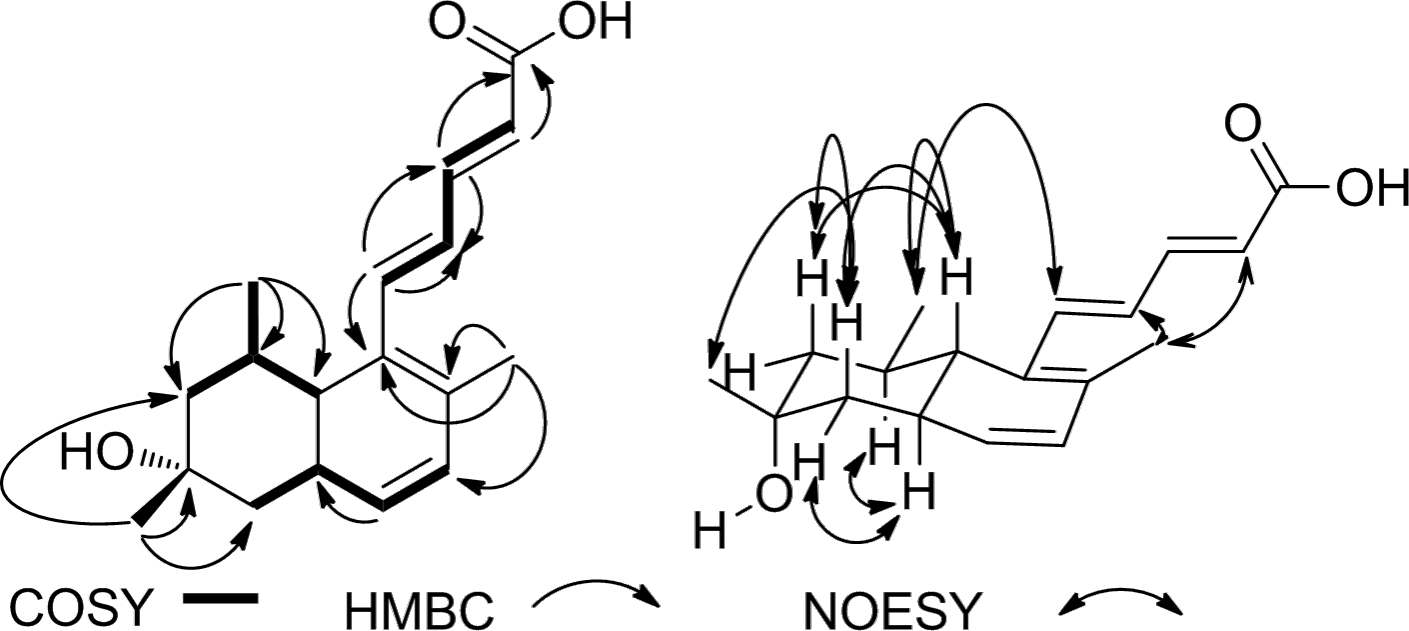
HMBC, COSY and NOESY correlations of compound **4**.

The molecular formula of compound **4**, C_18_H_24_O_3_ was determined based on its NMR and HRMS-ESI data which gave a pseudo-molecular ion at *m*/*z* 311.1630 (calcd for [M + Na]^+^: 311.1623). This composition required seven double bond equivalents. It differed from compound **3** by two hydrogen atoms suggesting one additional carbon–carbon double bond. This assumption was justified by the presence of four olefinic signals (δ_H_ 5.74 (dd, *J* = 2.2, 9.2 Hz, H-13)/δ_C_ 137.3, δ_H_ 5.97 (dd, *J* = 2.8, 9.2, H-14)/δ_C_ 131.0, δ_C_ 133.4 (C-6), and 133.8 (C-15)) in the 1D NMR spectra. The two latter signals were attributed to a tetrasubstituted olefin which was unique for compound **4**, along with only one CH_3_ group appearing as a doublet at δ_H_ 0.98 (d, *J* = 6.2 Hz, H-18)/δ_C_ 23.4. The second CH_3_ group observed as a doublet in compounds **1**–**3** appeared in the ^1^H NMR spectrum of **4** as a singlet (δ_H_ 1.20, H-17) and showed a HMBC correlation with the quaternary carbon at δ_C_ 70.1 (C-10) (Figure 4). As in compounds **1**–**3**, resonances of a (2*E*,4*E*)-penta-2,4-dienoic acid moiety as well as three additional CH groups, two CH_2_ groups and one CH_3_ for the completion of the decalin part were observed.

**Figure 5:**
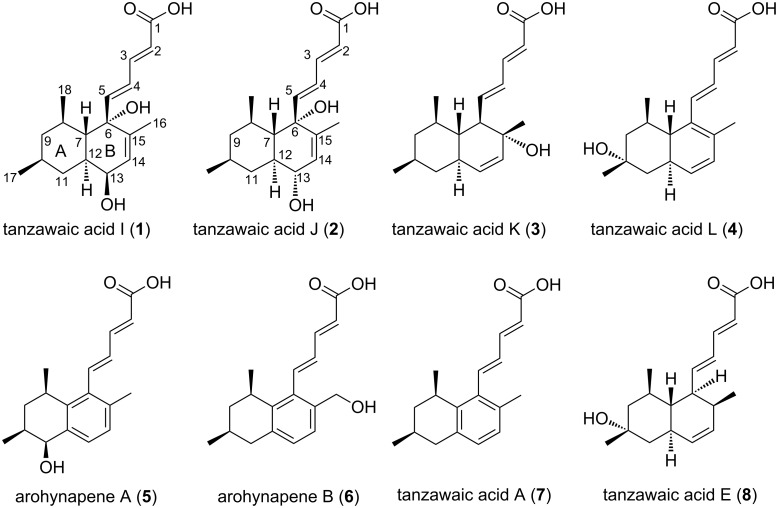
Structures of the isolated compounds.

The relative configuration of the stereogenic centers was determined by NOESY (Figure 4). Correlations were observed between H-7 (δ_H_ 1.56) and H-9_ax_ (δ_H_ 1.06), H-11_ax_ (δ_H_ 1.33), and the C-18 methyl protons (δ_H_ 0.98). An additional correlation was found between H-11_ax_ (δ_H_ 1.33) and the C-17 methyl protons (H-17, δ_H_ 1.20) suggesting an equatorial orientation of the latter. From the complete spectral assignment, the structure of compound **4** was established as shown in Figure 5. The fourth member of the series was consequently named tanzawaic acid L (Figure 5).

**Figure 6:**
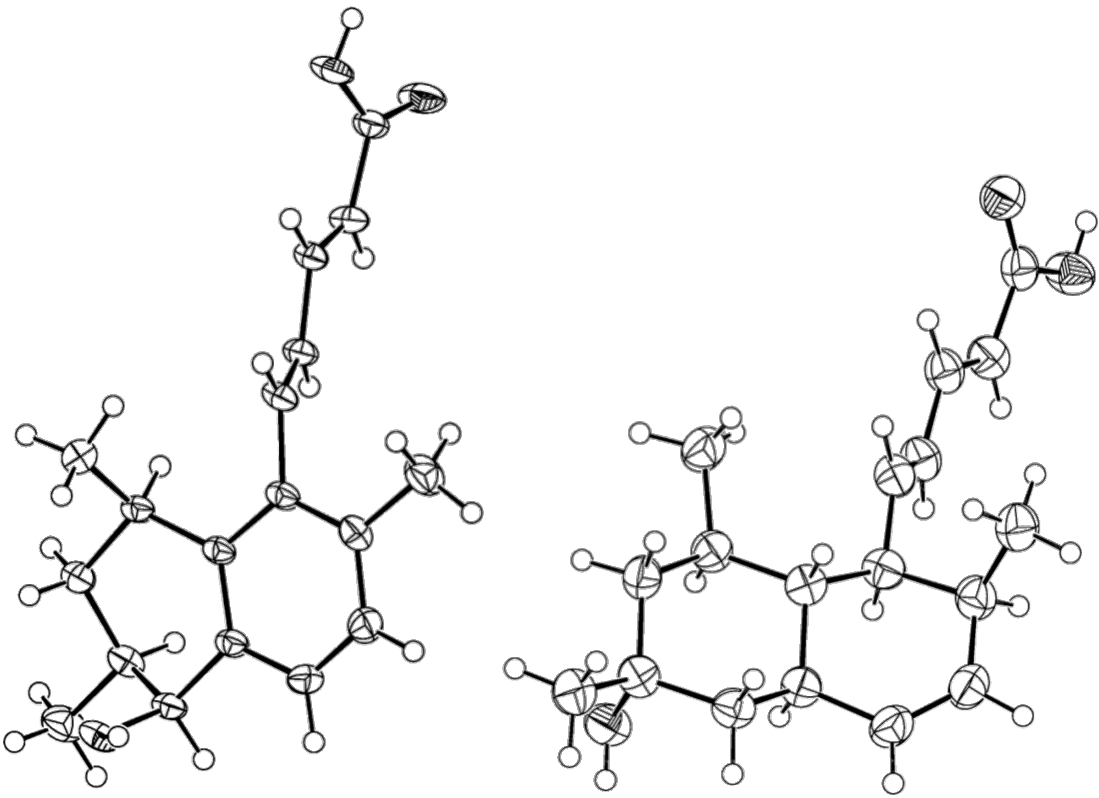
Crystal structures (ORTEP plot) of arohynapene A (**5**, left) and tanzawaic acid E (**8**, right). Thermal ellipsoids are drawn at the 50% probability level.

Along with compounds **1**–**4**, four known polyketides were isolated and identified (Figure 5) as arohynapene A (**5**) [3], arohynapene B (**6**) [3], tanzawaic acid A (**7**) [1], and tanzawaic acid E (**8**) [2]. Their structures were established based on NMR data and by comparison with those reported in the literature. The structures of arohynapene A and tanzawaic acid E were furthermore supported by X-ray crystallography (Figure 6). In the latter case, the absolute configuration could be determined even though no heavy atom is present in the unit cell. The result confirms the synthetic work of Arimoto et al. who determined the configuration of tanzawaic acid A to be 8*R*,10*S* [4] and suggests the other tanzawaic acids to have the same absolute configuration.

Tanzawaic acids were found in different *Penicillium* species, especially tanzawaic acid A which seems to be omnipresent in *Penicillium citrinum* [1–2]. Tanzawaic acids E and F were isolated from a marine-derived *Penicillium steckii* and were detected in at least 16 culture collection strains of the same species in a secondary metabolite study [2].

Recently, El-Neketi et al. [5] described the isolation of two new tanzawaic acids G and H, as well as tanzawaic acids B and F and arohynapene D from an endophytic *Penicillium citrinum* species. Therefore, *Penicillium* sp. IBWF104-06 appears to be related by its secondary metabolites to the aforementioned strains which do belong to the *Penicillium* section *Citrina* [6]. Tanzawaic acid E differs from tanzawaic acid L by two more hydrogen atoms at C-6 and C-15 and has the same elemental composition as tanzawaic acid K, the structural difference being the position of the OH group located at C-15 of tanzawaic acid K instead of C-10 in tanzawaic acid E [2]. Tanzawaic acids I and J have an 18 amu lower mass than hynapene A [7]. In contrast to the latter compound, both tanzawaic acids contain a double bond at C-14/15 instead of an OH group at C-14.

**Biological activity.** The isolated compounds were tested for their bioactivity in a series of antimicrobial and antiproliferative assays. We found that apart from compounds **1** and **2**, the other isolates (**3**–**8**) inhibited the conidial germination in *Magnaporthe oryzae* at concentrations of 50 µg/mL or less (Table 3) whereas the germination of the grey mold *Botrytis cinerea* and the potato blight caused by the oomycete *Phytophthora infestans* were not affected at comparable concentrations. Even though several biological activities have been found for the known compounds **5**–**8**, an inhibition of fungal spore germination of *Magnaporthe oryzae* was not reported to date. The structurally related hynapenes B and C showed antimicrobial activity against *Pyricularia oryzae* [8] whereas arohynapenes A, B, and D [3,9] were not active in agar diffusion assays. However, arohynapenes A and B inhibited the spore germination at 25 µg/mL (IC_90_). In addition, tanzawaic acid A (**7**) showed weak activity in agar diffusion assays at 50 µg/filter dics against *Bacillus brevis*, *Mucor miehei*, and *Paecilomyces variotii*, as well as cytotoxic effects against HeLaS3 cells at a concentration of 50 µg/mL. However, none of the other compounds showed bioactivity in these assays up to 50 µg/mL.

**Table 3:** Inhibition of germination in *Magnaporthe oryzae* (IC_90_ represents the concentration at which 90% inhibition of conidial germination was observed).

Compound	*M. oryzae* IC_90_ [µg/mL]

tanzawaic acid I (**1**)	–
tanzawaic acid J (**2**)	–
tanzawaic acid K (**3**)	25
tanzawaic acid L (**4**)	50
arohynapene A (**5**)	25
arohynapene B (**6**)	25
tanzawaic acid A (**7**)	10
tanzawaic acid E (**8**)	25

## Experimental

**General experimental procedures.** Optical rotations were measured on a Perkin Elmer polarimeter model 241 at 546 and 578 nm and were extrapolated to 589 nm using Drude’s equation [10]. ^1^H, ^13^C and 2D NMR spectra were recorded on a Bruker AVANCE III 600 MHz spectrometer equipped with a 5 mm TCI cryogenic inverse probe head (*z*-gradient) using standard pulse sequences. APCI-MS spectra were measured from a solution of the analyte in MeCN/H_2_O with a Hewlett Packard MSD 1100 using an evaporator temperature of 400 °C and a drying gas temperature of 350 °C at a flow of 6 L/h (N_2_). In positive ionization mode, the capillary voltage amounted to 3.5 kV, the corona discharge current was 4 µA. In negative ionization mode, the capillary voltage amounted to 2.2 kV, the corona discharge current was 6 µA. HRMS-ESI data were recorded on a Q-ToF ULTIMA-III (Waters) equipped with a LockSpray-interface. A Bruker IFS48 FTIR spectrometer and a Perkin-Elmer Lambda-16 spectrophotometer were used to measure the IR and UV spectra, respectively.

**Producing organism.** The producing fungus IBWF104-06 was isolated from a soil sample. The strain was determined as a *Penicillium* species by morphology. Strain IBWF104-06 has been deposited in the culture collection of the Institute of Biotechnology and Drug Research (IBWF e.V.), Kaiserslautern, Germany. For maintenance, the fungus was grown on agar slants on YMG agar (yeast extract 4.0 g/L, malt extract 10 g/L, glucose 10 g/L, the pH value was adjusted to 5.5 before sterilisation). Solid media contained 2.0% agar.

**Fermentation and isolation.** The fermentation was carried out according to the method described by Schüffler et al. [11]. The fungus was grown in YMG medium in a 20 L fermenter (Braun, Melsungen) at 22–24 °C with agitation (120 rpm) and aeration (3 L/min). For inoculation, a well-grown shake culture (YMG medium, 250 mL) was used. During fermentation, the bioactive principles were quantified by HPLC and 13 days after inoculation the production was pronounced and the fermentation was stopped. The culture fluid (14.5 L) was separated from the mycelium by filtration and extracted with ethyl acetate (10 L). The solvent was evaporated and the oily crude extract (2.2 g) was applied onto a column filled with silica gel (Merck 60, 0.063–0.2 mm). Elution with a mixture of cyclohexane/ethyl acetate (3:2 v/v) yielded the oily fraction 1 (700 mg), 2:3 cyclohexane/ethyl acetate eluted oily fraction 2 (560 mg), and 1:4 cyclohexane/ethyl acetate eluted oily fraction 3 (100 mg). Further work-up of fraction 1 by solid-phase extraction (Macherey-Nagel, Chromabond C18ec) with 1:1 acetonitrile/water generated intermediate A (600 mg) and with 1:4 acetonitrile/water intermediate B (50 mg). Preparative HPLC of intermediate A (Waters SunFire, Prep C18 OBD, 5 µm, 19 × 250 mm, 17 mL/min, isocratic conditions, 1:1 acetonitrile/0.1% formic acid) resulted in arohynapene A (**5**, 173 mg, *t*_R_ 9 min) and tanzawaic acid E (**8**, 143 mg, *t*_R_ 13 min). Preparative HPLC of intermediate B resulted in tanzawaic acid A (**7**, 20 mg, *t*_R_ 24 min; Waters SunFire, Prep C18 OBD, 5 µm, 19 × 250 mm, 17 mL/min, isocratic conditions, 13:7 acetonitrile/0.1% formic acid). Work-up of fraction 2 by preparative HPLC (Waters SunFire, Prep C18 OBD, 5 µm, 19 × 250 mm, 17 mL/min, isocratic conditions, 11:9 acetonitrile/0.1% formic acid) generated tanzawaic acid K (**3**, 54 mg, *t*_R_ 16.5 min) and intermediate C (*t*_R_ 7–13 min, 320 mg). Intermediate C yielded tanzawaic acid L (**4**, 29 mg, *t*_R_ 25.5 min) and arohynapene B (**6**, 14 mg, *t*_R_ 31 min) by a second preparative HPLC (Agilent PrepHT, Zorbax, XDB-C8, 21.2 × 150 mm, 5 µm, 21 mL/min, isocratic conditions, 7:13 acetonitrile/0.1% formic acid). Fraction 3 was applied onto a solid-phase extraction column (Macherey-Nagel, Chromabond C18ec) and extraction with 2:3 acetonitrile/0.1% formic acid gave intermediate D (87 mg) which was used for preparative HPLC (Waters SunFire, Prep C18 OBD, 5 µm, 19 × 250 mm, 16 mL/min, isocratic conditions, 2:3 acetonitrile/0.1% formic acid) to yield tanzawaic acid I (**1**, 7.7 mg, *t*_R_ 9 min) and tanzawaic acid J (**2**, 12.6 mg, *t*_R_ 10.5 min).

**Bioactivity assays.** Antimicrobial activities against bacteria and fungi were determined in the agar plate diffusion assay as described previously [12]. Cytotoxicity was assayed as described previously [13]. The cell line HelaS3 was grown in DMEM medium (Invitrogen). The medium was supplemented with 10% heat-inactivated fetal calf serum (Invitrogen), 65 mg/L of penicillin G and 100 mg/L of streptomycin sulfate. The viability was evaluated by microscopy after 72 h. The spore germination was tested with *M. oryzae* as described previously [14]. This method was adapted for the spore germination assay with *Phytophthora infestans* and *Botrytis cinerea*.

**Tanzawaic acid I (1).** Yellow oil; UV (MeOH) (λ_max_, log ε): 261 (4.45); [α]_D_^20^ −262 (0.4, MeOH); IR (ν, cm^–1^): 3414, 2950, 2923, 1689, 1640, 1457, 1380, 1248; HRMS-ESI *m/z*: [M + Na]^+^ calcd for C_18_H_26_O_4_Na, 329.1729; found, 329.1744; APCI-MS (neg) *m/z*: 305.1 [M − H]^−^; APCI-MS (pos) *m/z*: 289.2 [M – H_2_O + H]^+^, 271.2 [M – 2H_2_O + H]^+^.

**Tanzawaic acid J (2).** Yellow oil; UV (MeOH) (λ_max_, log ε): 261 (4.45); [α]_D_^20^ +73.7 (0.4, MeOH); IR (ν, cm^–1^): 3369, 2947, 2835, 1638, 1451, 1032; HRMS-ESI *m/z*: [M + Na]^+^ calcd for C_18_H_26_O_4_Na, 329.1729; found, 329.1744; APCI-MS (neg) *m/z*: 305.1 [M − H]^−^; APCI-MS (pos) *m/z*: 289.2 [M – H_2_O + H]^+^, 271.2 [M – 2H_2_O + H]^+^.

**Tanzawaic acid K (3).** Yellow oil; UV (MeOH) (λ_max_, log ε): 257 (4.59); [α]_D_^20^ −20.1 (0.38, MeOH); IR (ν, cm^–1^): 3432, 2909, 1688, 1638, 1456, 1275; HRMS-ESI *m/z*: [M + Na]^+^ calcd for C_18_H_26_O_3_Na, 313.1780; found, 313.1788; APCI-MS (neg) *m/z*: 289.1 [M − H]^−^; APCI-MS (pos) *m/z*: 273.2 [M – H_2_O + H]^+^.

**Tanzawaic acid L (4).** Yellow oil; UV (MeOH) (λ_max_, log ε): 255 (3.70), 286 (3.68); [α]_D_^20^ −126.7 (0.32, MeOH); IR (ν, cm^–1^): 3409, 2961, 2965, 1698, 1638, 1377, 1247; HRMS-ESI *m/z*: [M + Na]^+^ calcd for C_18_H_24_O_3_Na, 311.1623; found, 311.1630; APCI-MS (neg) *m/z*: 287.1 [M − H]^−^; APCI-MS (pos) *m/z*: 271.1 [M – H_2_O + H]^+^, 253.1 [M – 2H_2_O + H]^+^.

CCDC 962065 and 962066 contain crystallographic data for arohynapene A and tanzawaic acid E, respectively. A copy of the data can be obtained free of charge from the Cambridge Crystallographic Data Centre, on application to CCDC, 12 Union Road, Cambridge CB2 1EZ, UK, (fax: +44-(0)1223-336033 or e-mail:deposit@ccdc.cam.ac.uk).

## Supporting Information

File 1 NMR spectra and crystallographic data.
