# Supplementary material for: Tanzawaic acids I–L: Four new polyketides from Penicillium sp. IBWF104-06
Source: Beilstein J Org Chem. 2014 Jan 22;10:251–8. doi: 10.3762/bjoc.10.20 (PMC3943810; doi:10.3762/bjoc.10.20)
Supplement: File 1 — NMR spectra and crystallographic data. [file Beilstein_J_Org_Chem-10-251-s001.pdf]

Supporting Information

for

Tanzawaic acids I–L: Four new polyketides from *Penicillium* sp. IBWF104-06

Louis P. Sandjo[1], Eckhard Thines[2,3], Till Opatz*[,1] and Anja Schüffler*[,2]

Address: [1]Johannes-Gutenberg-University Mainz, Institute of Organic Chemistry, Duesbergweg 10-14, D-55128 Mainz, Germany, [2]Institute of Biotechnology and Drug Research, Erwin-Schroedinger-Strasse 56, D-67663, Kaiserslautern, Germany and [3]Johannes-Gutenberg-University Mainz, Institute of Biotechnology and Drug Research, Duesbergweg 10–14, D-55128 Mainz, Germany

Email: Anja Schüffler* - schueffler@ibwf.de; Till Opatz* - opatz@uni-mainz.de

*Corresponding author

NMR spectra and crystallographic data

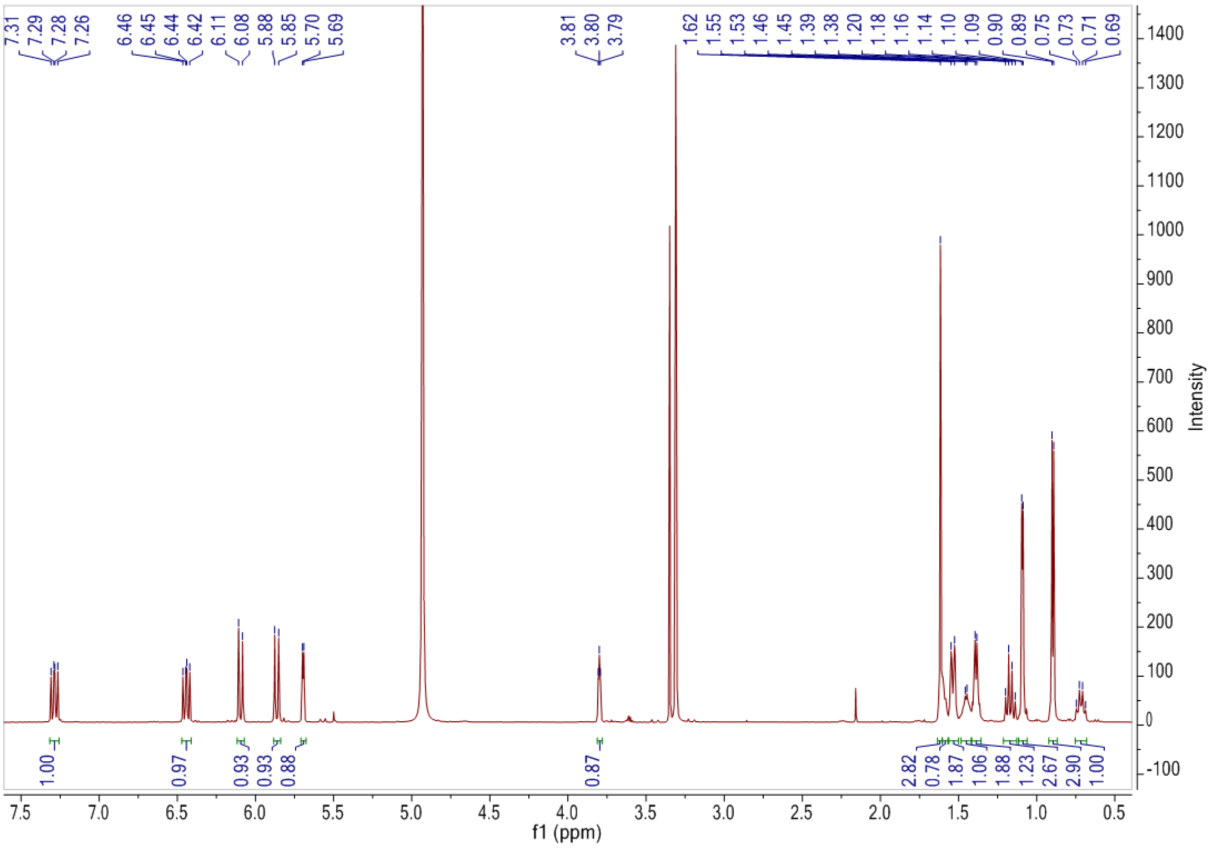

Figure S1: ^{1}H NMR spectrum of tanzawaic acid I (**1**) (CD_3OD).

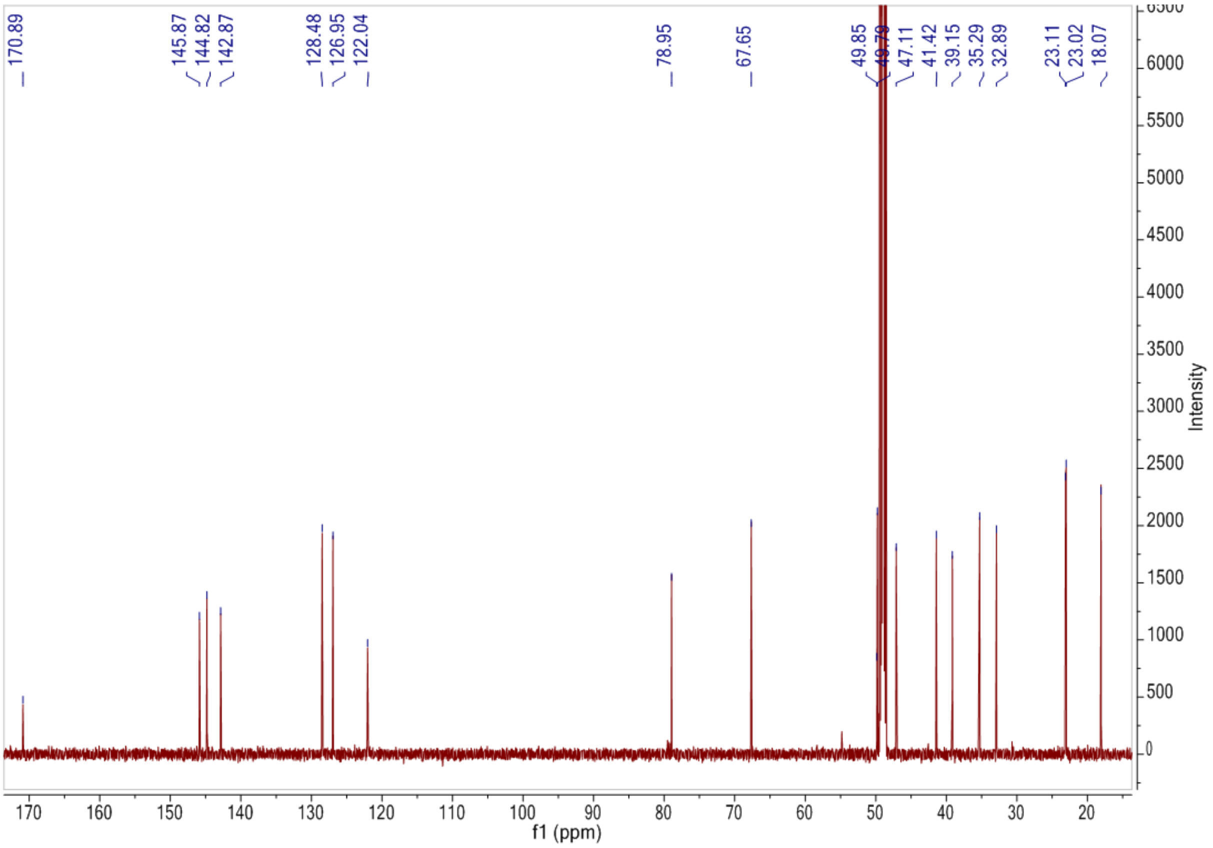

Figure S2: ^{13}C NMR spectrum of tanzawaic acid I (**1**) (CD_3OD).

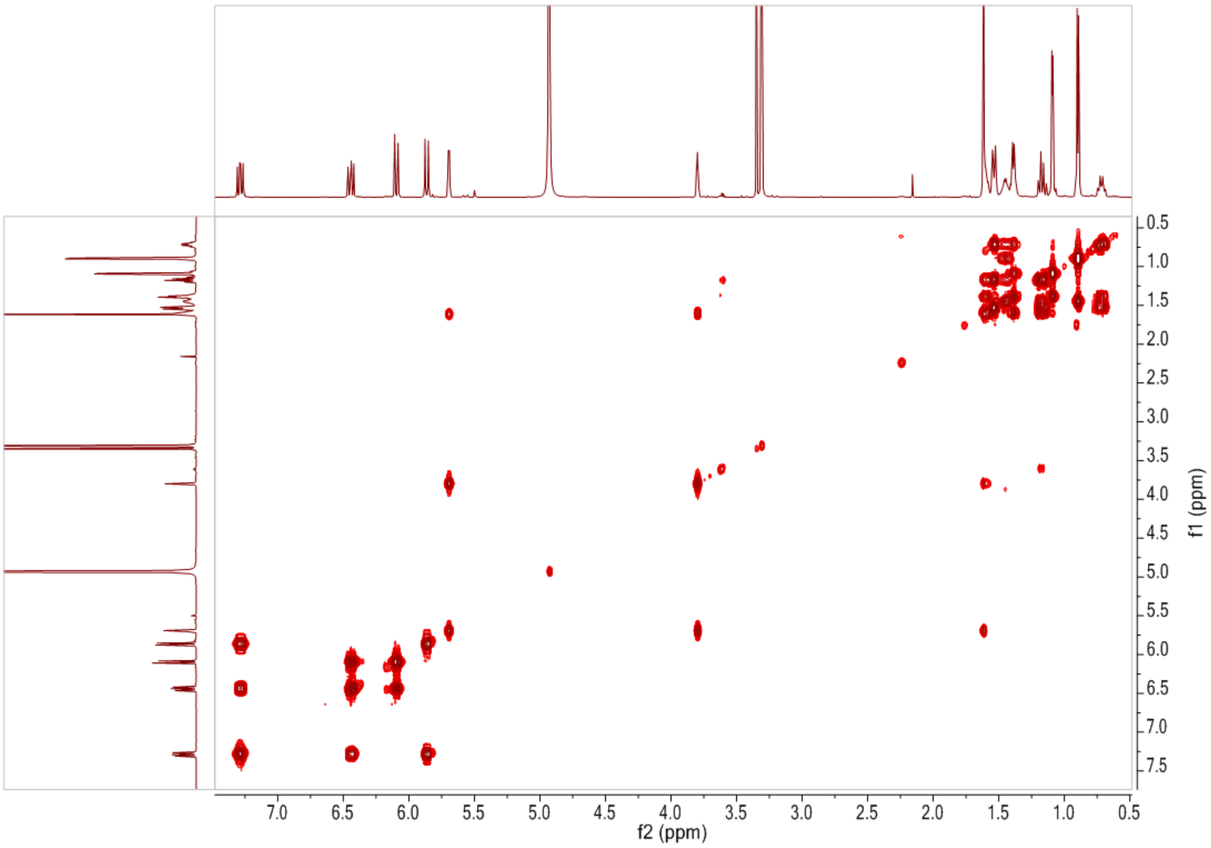

Figure S3: COSY spectrum of tanzawaic acid I (**1**) (CD_3OD).

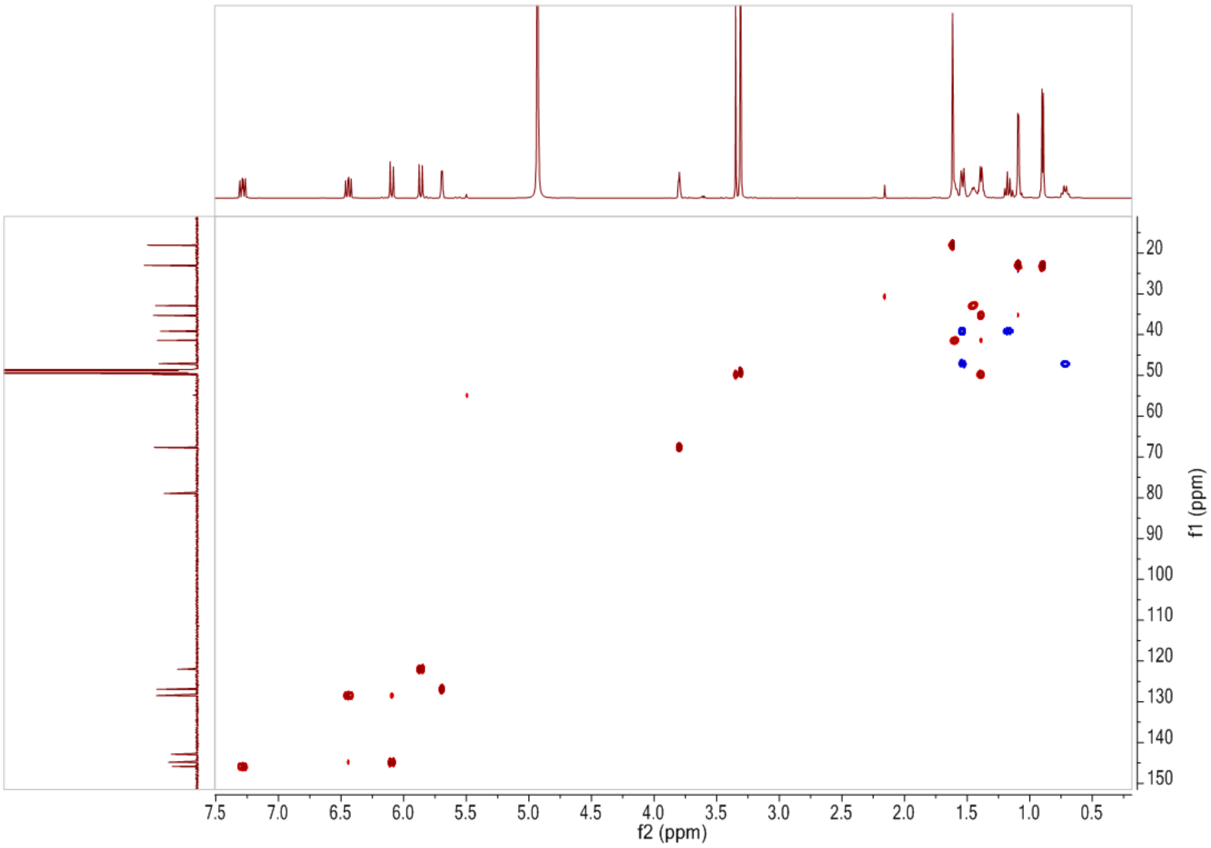

Figure S4: HSQC spectrum of tanzawaic acid I (**1**) (CD_3OD).

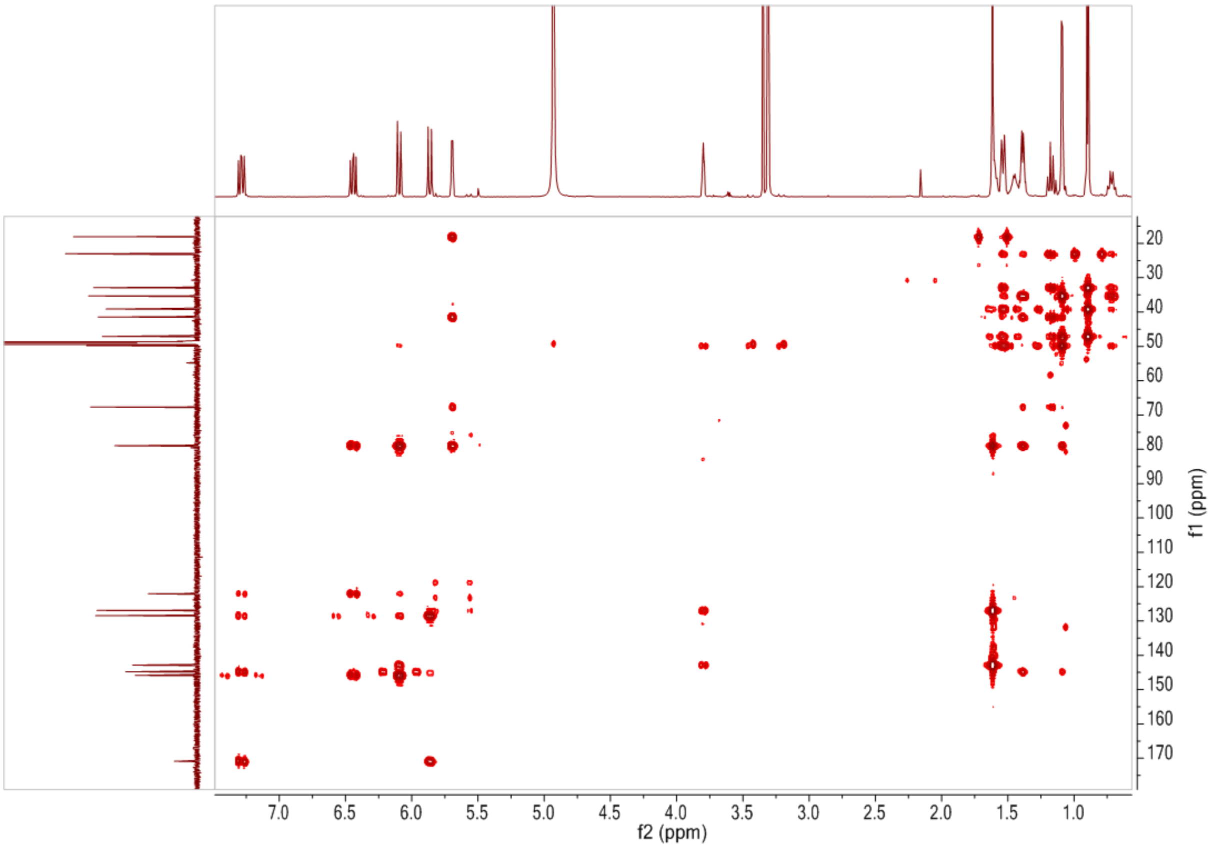

Figure S5: HMBC spectrum of tanzawaic acid I (**1**) (CD_3OD).

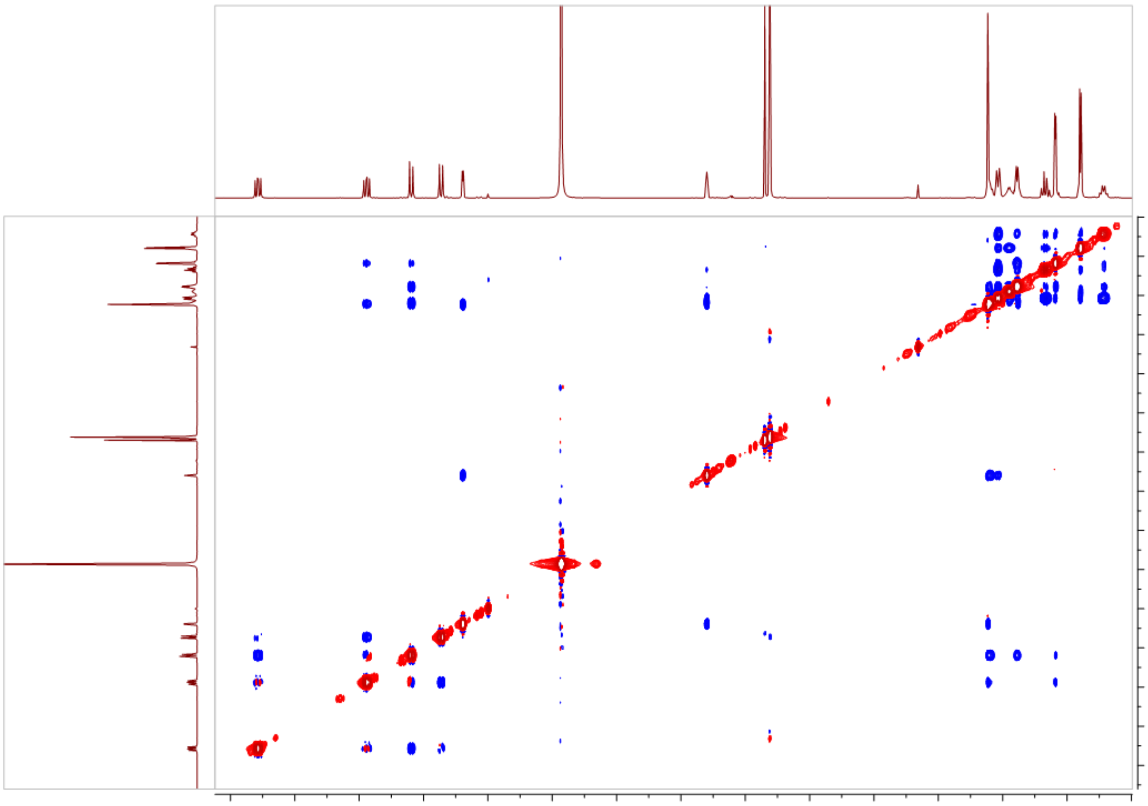

Figure S6: NOESY spectrum of tanzawaic acid I (**1**) (CD_3OD).

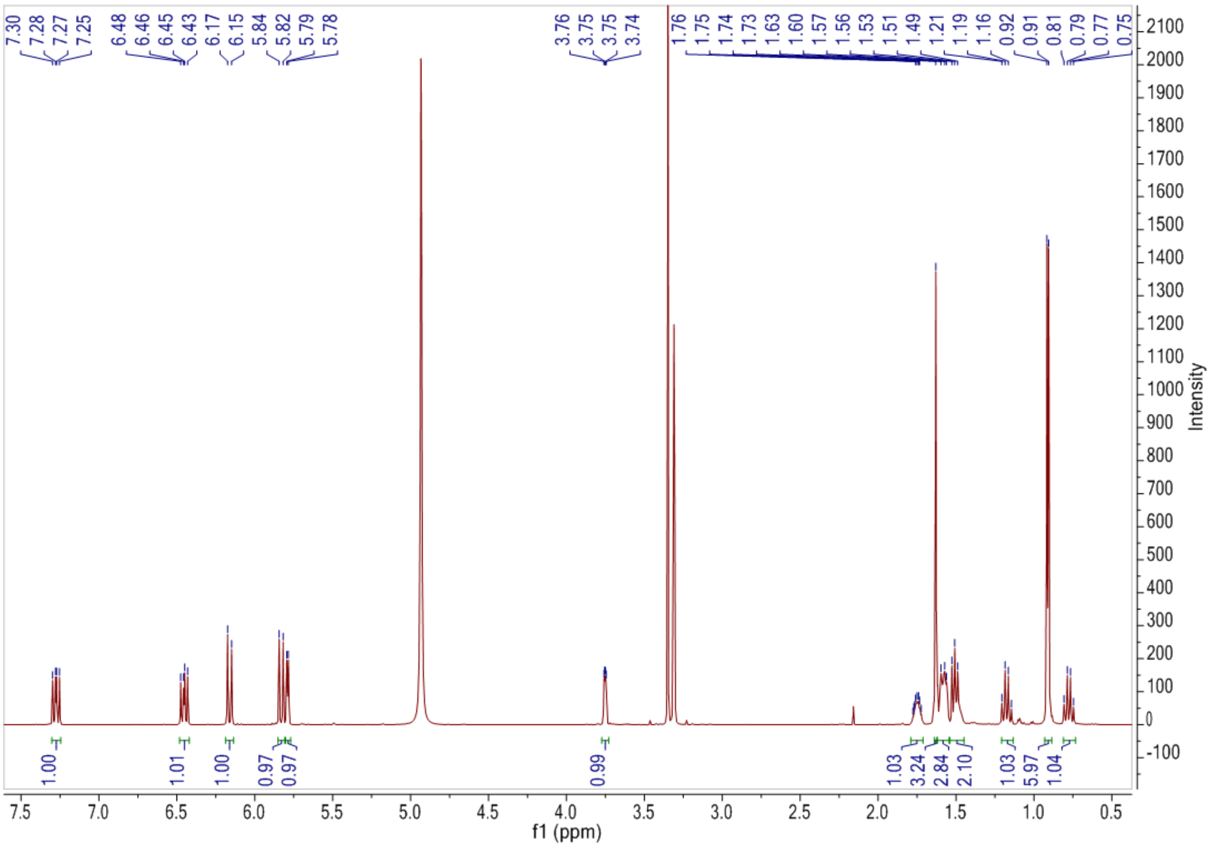

Figure S7: ^{1}H NMR spectrum of tanzawaic acid J (**2**) (CD_3OD).

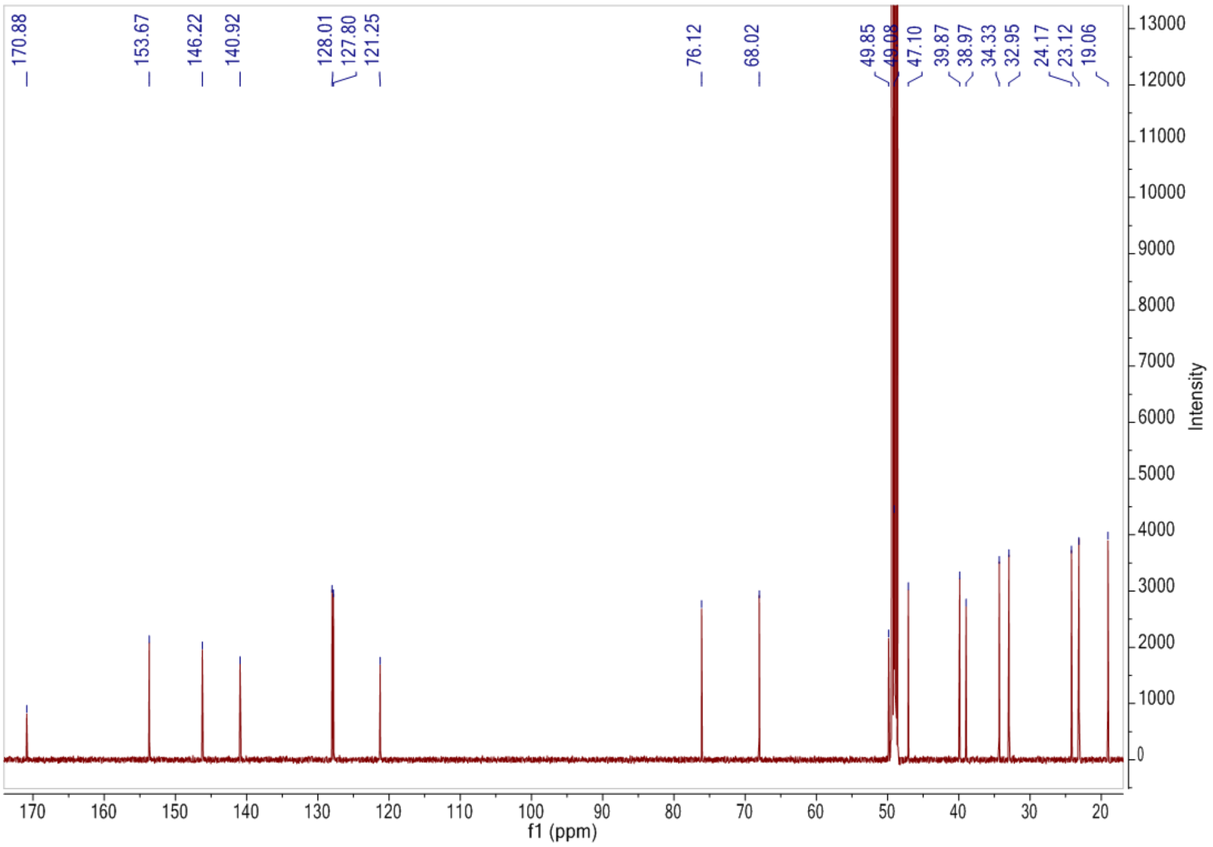

Figure S8: ^{13}C NMR spectrum of tanzawaic acid J (**2**) (CD_3OD).

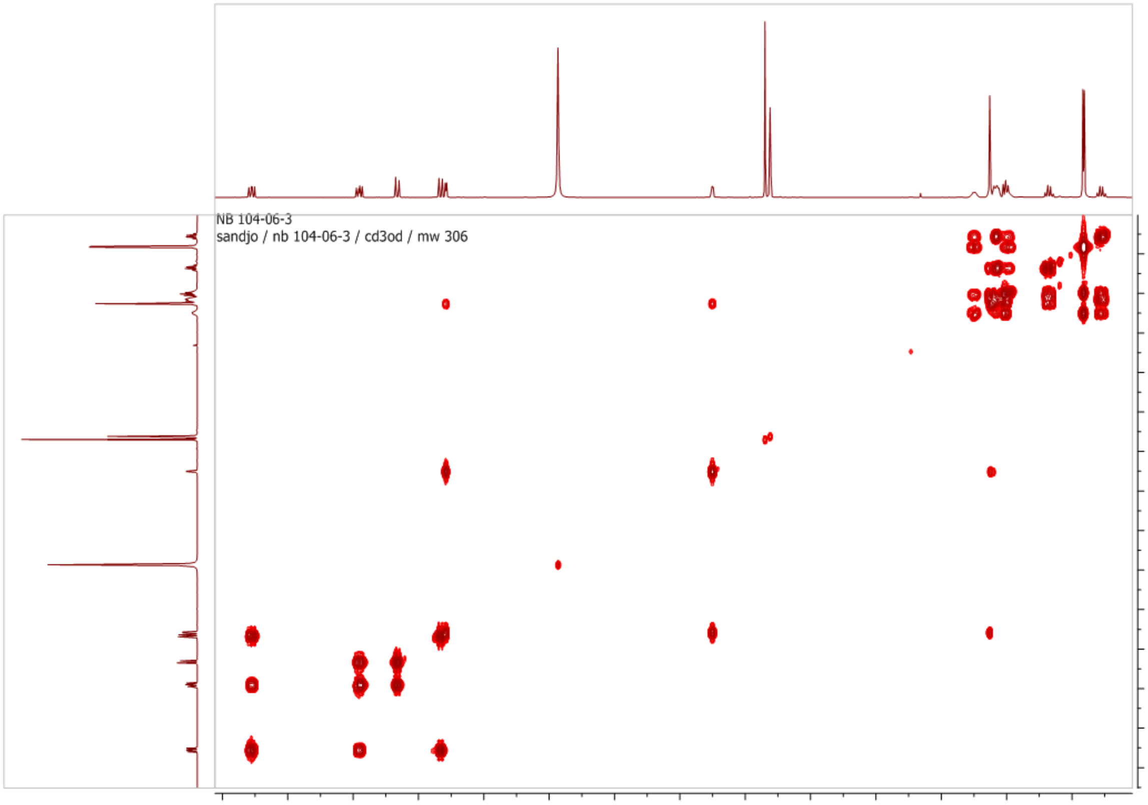

Figure S9: COSY spectrum of tanzawaic acid J (**2**) (CD_3OD).

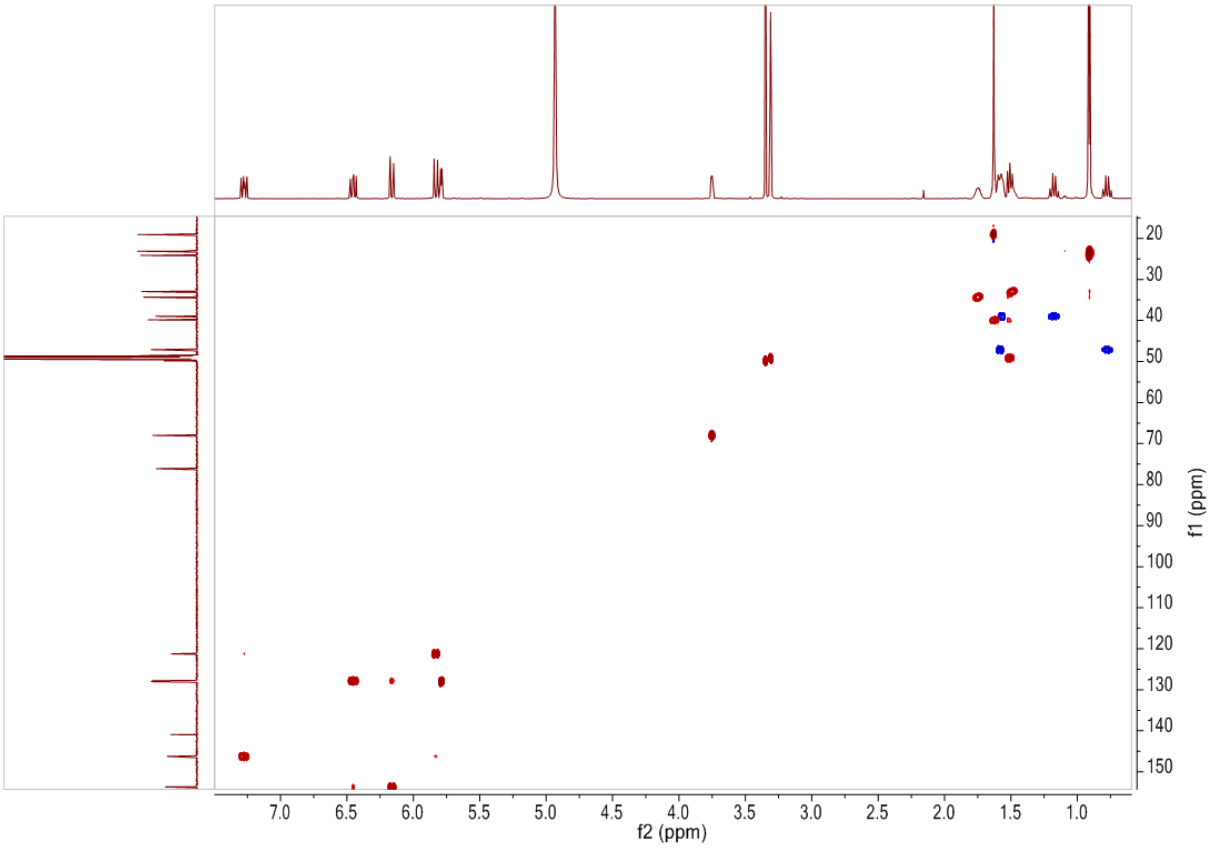

Figure S10: HSQC spectrum of tanzawaic acid J (**2**) (CD_3OD).

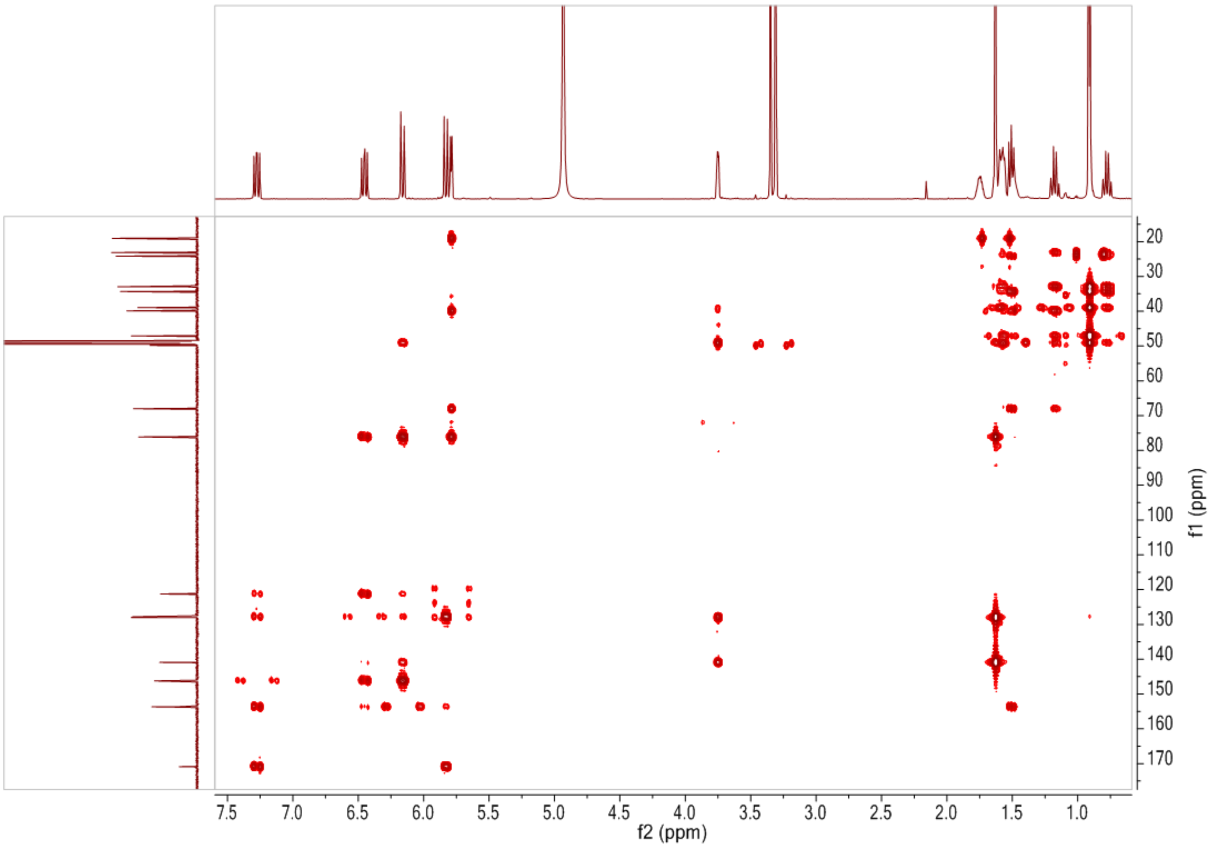

Figure S11: HMBC spectrum of tanzawaic acid J (**2**) (CD_3OD).

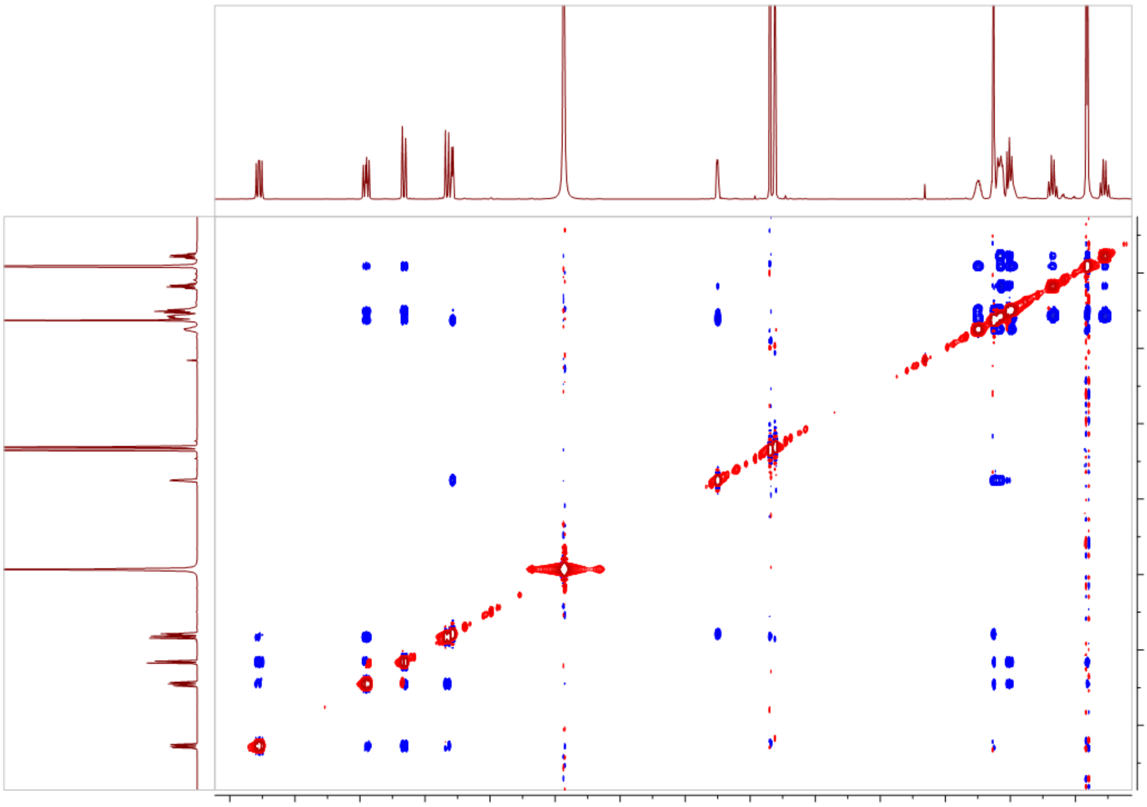

Figure S12: NOESY spectrum of tanzawaic acid J (**2**) (CD_3OD).

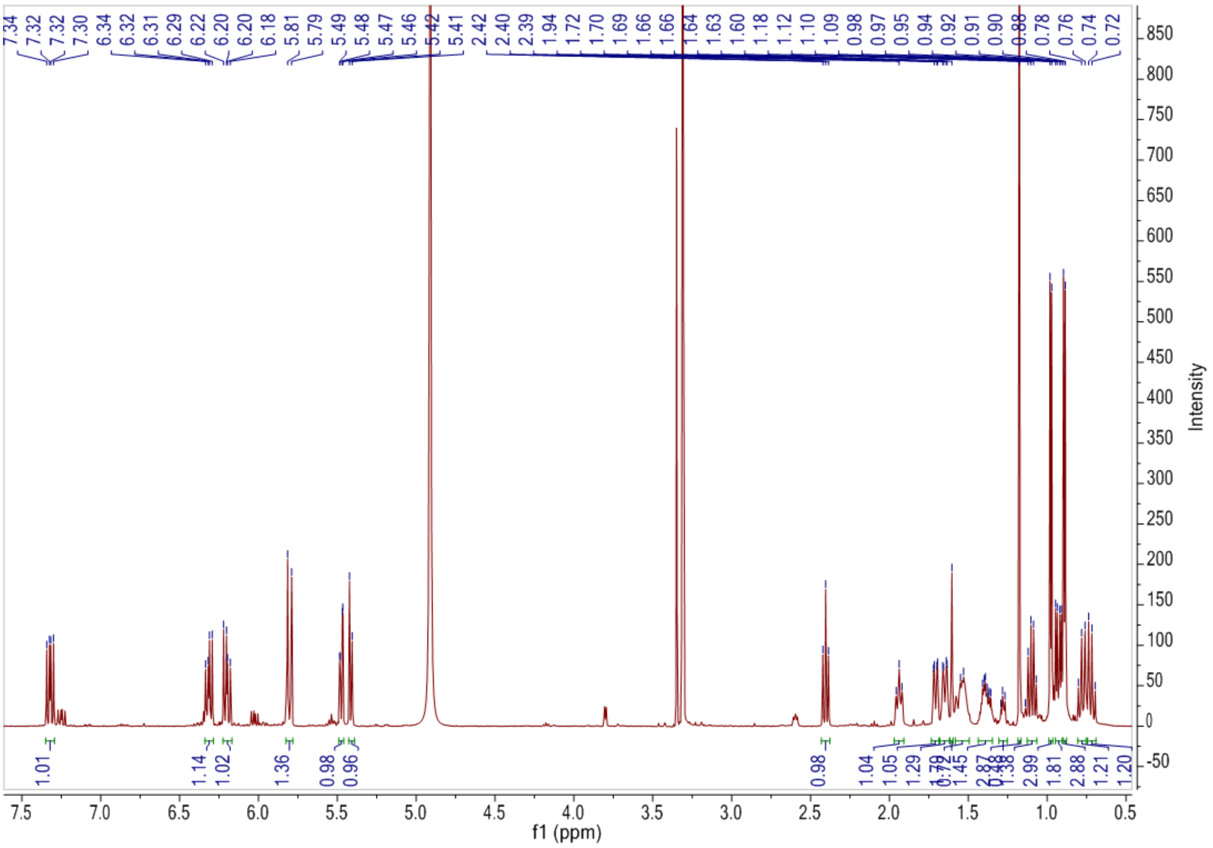

Figure S13: ^{1}H NMR spectrum of tanzawaic acid K (**3**) (CD_3OD).

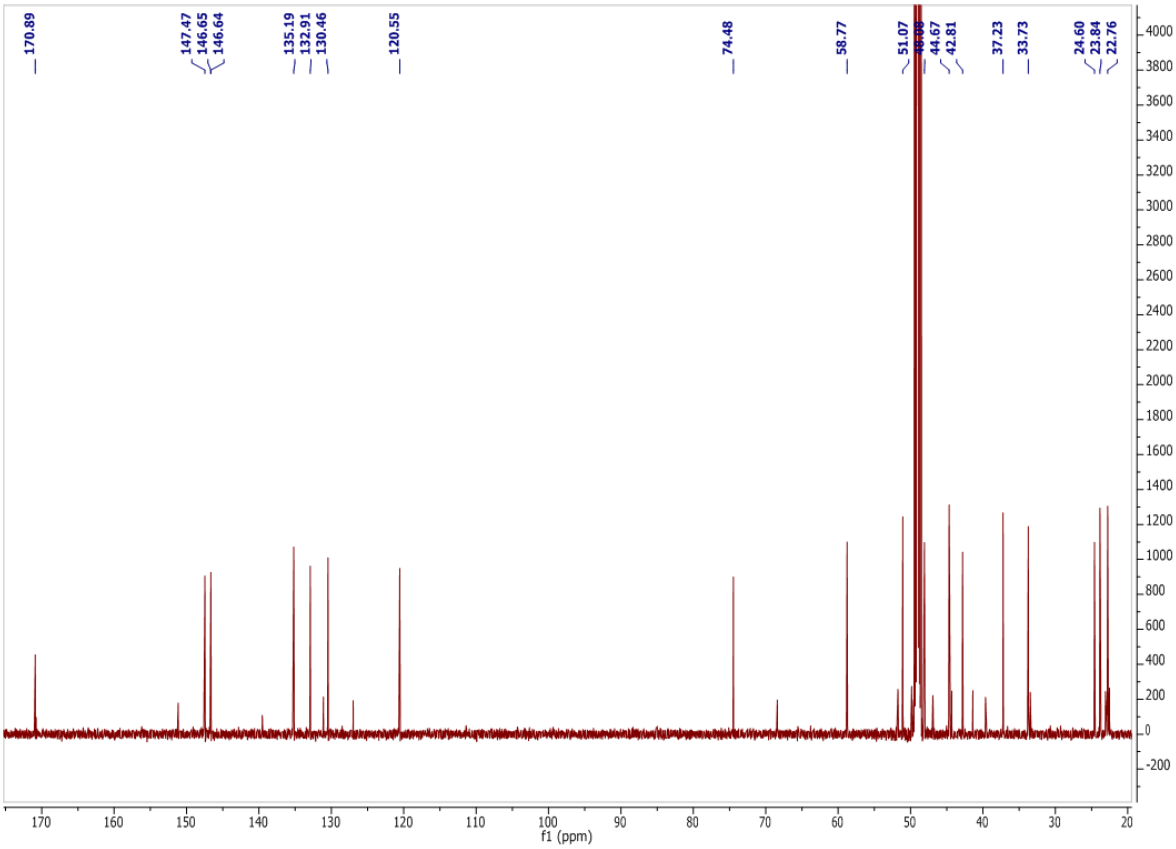

Figure S14: ^{13}C NMR spectrum of tanzawaic acid K (**3**) (CD_3OD).

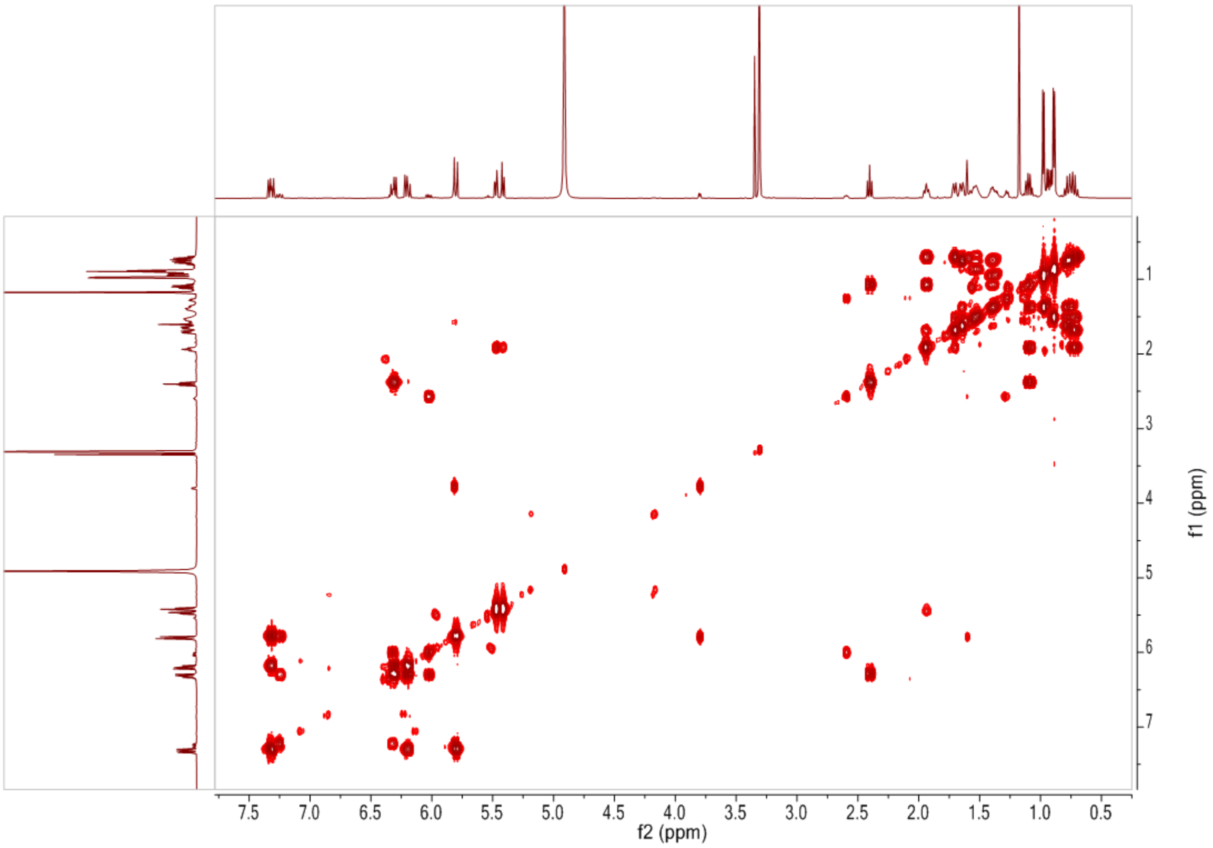

Figure S15: COSY spectrum of tanzawaic acid K (**3**) (CD_3OD).

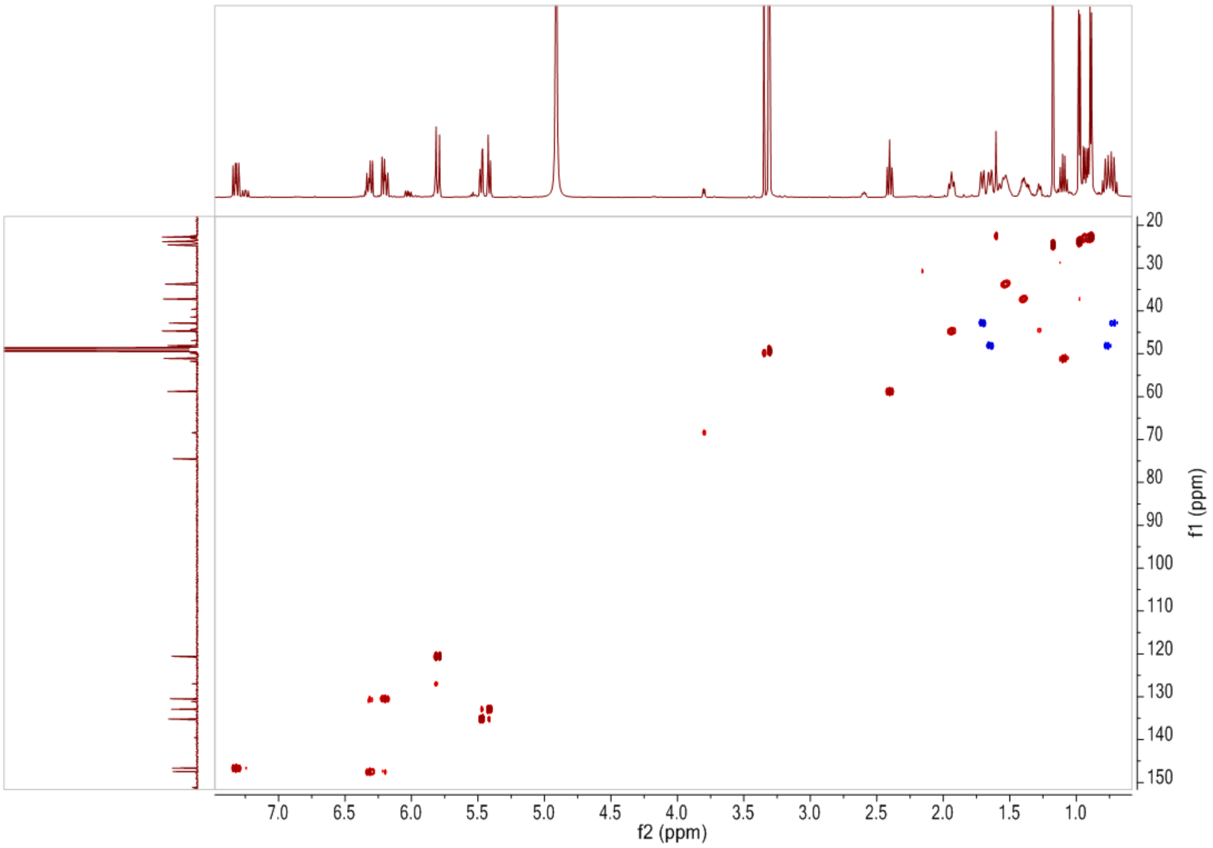

Figure S16: HSQC spectrum of tanzawaic acid K (**3**) (CD_3OD).

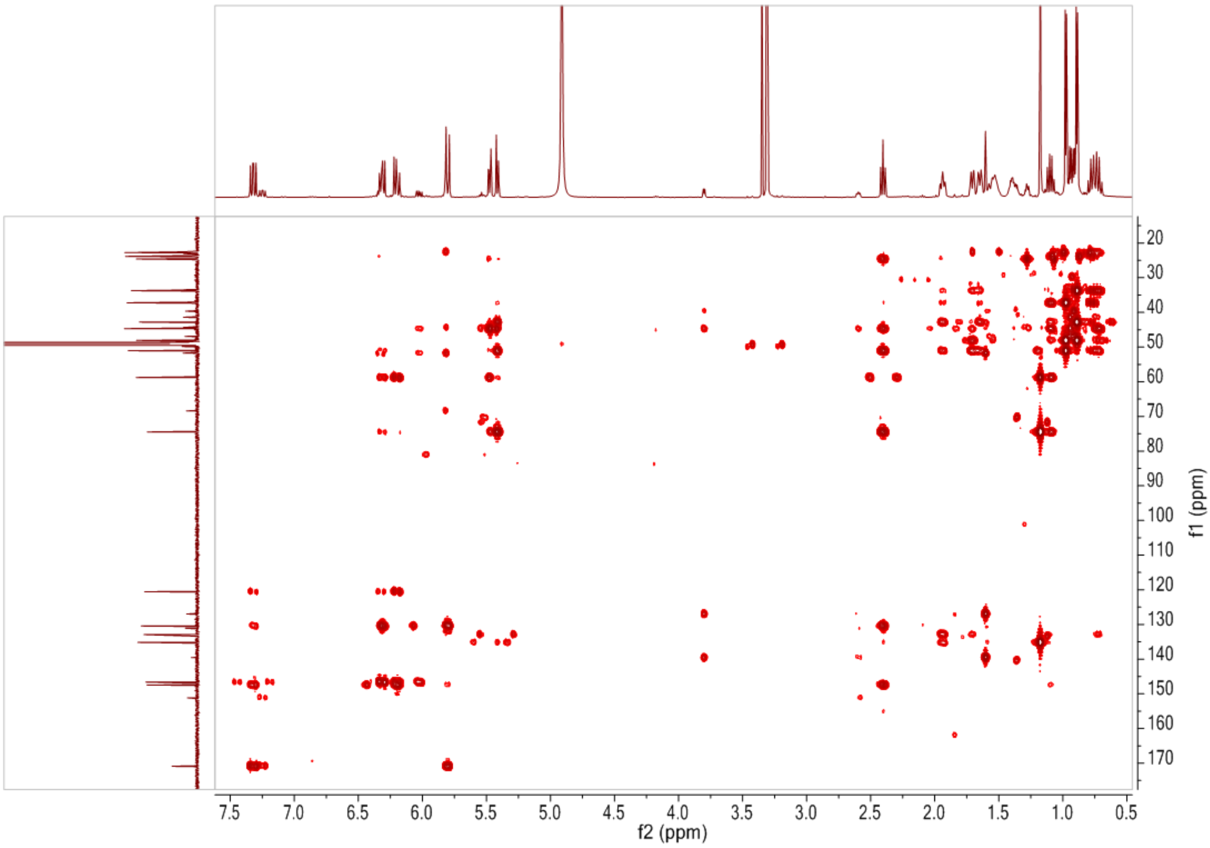

Figure S17: HMBC spectrum of tanzawaic acid K (**3**) (CD_3OD).

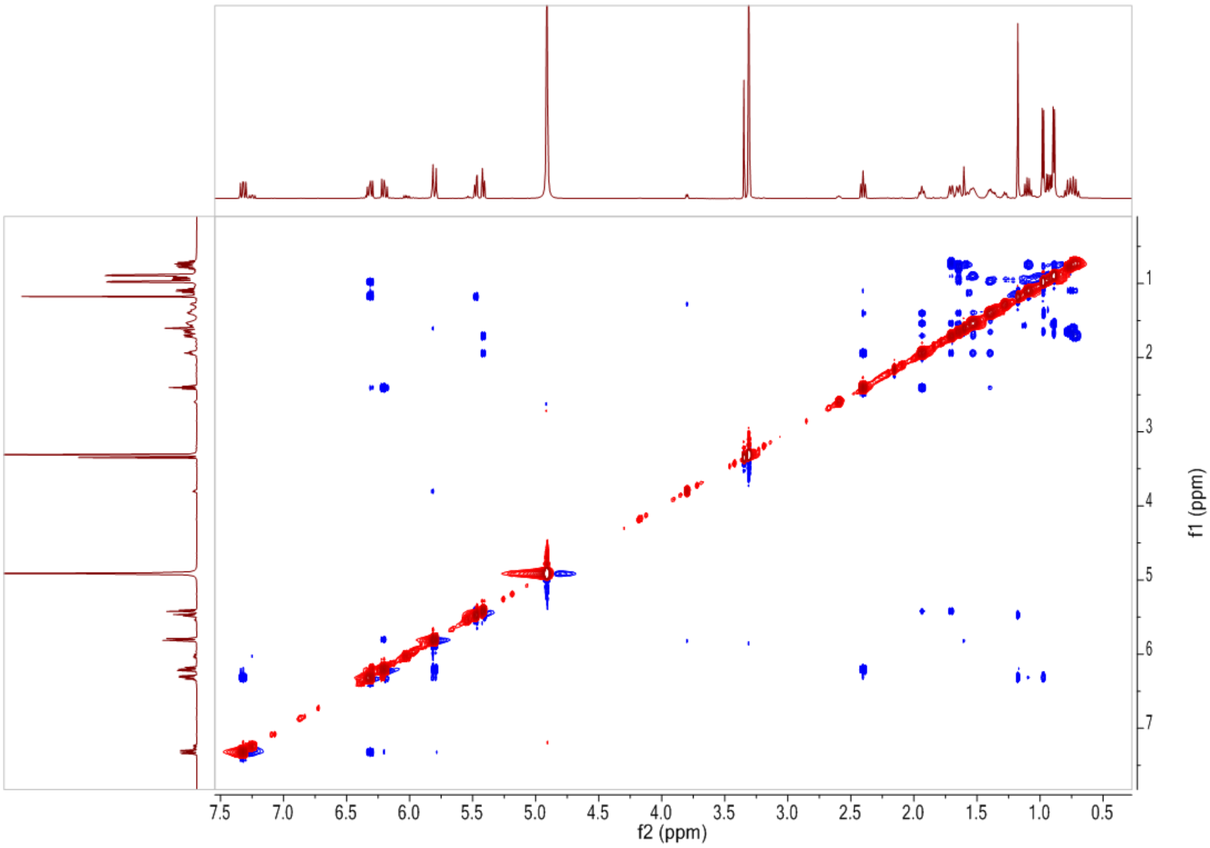

Figure S18: NOESY spectrum of tanzawaic acid K (**3**) (CD_3OD).

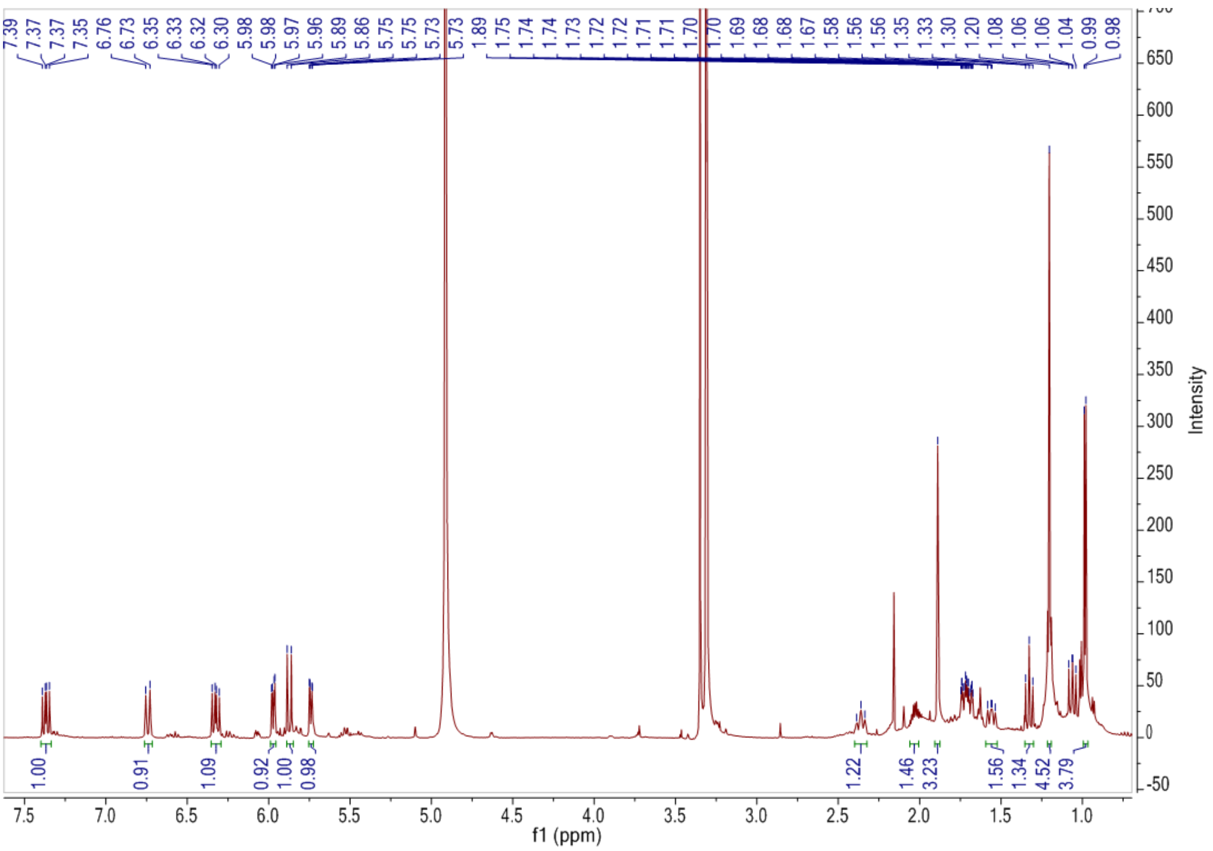

Figure S19: ^{1}H NMR spectrum of tanzawaic acid L (**4**) (CD_3OD).

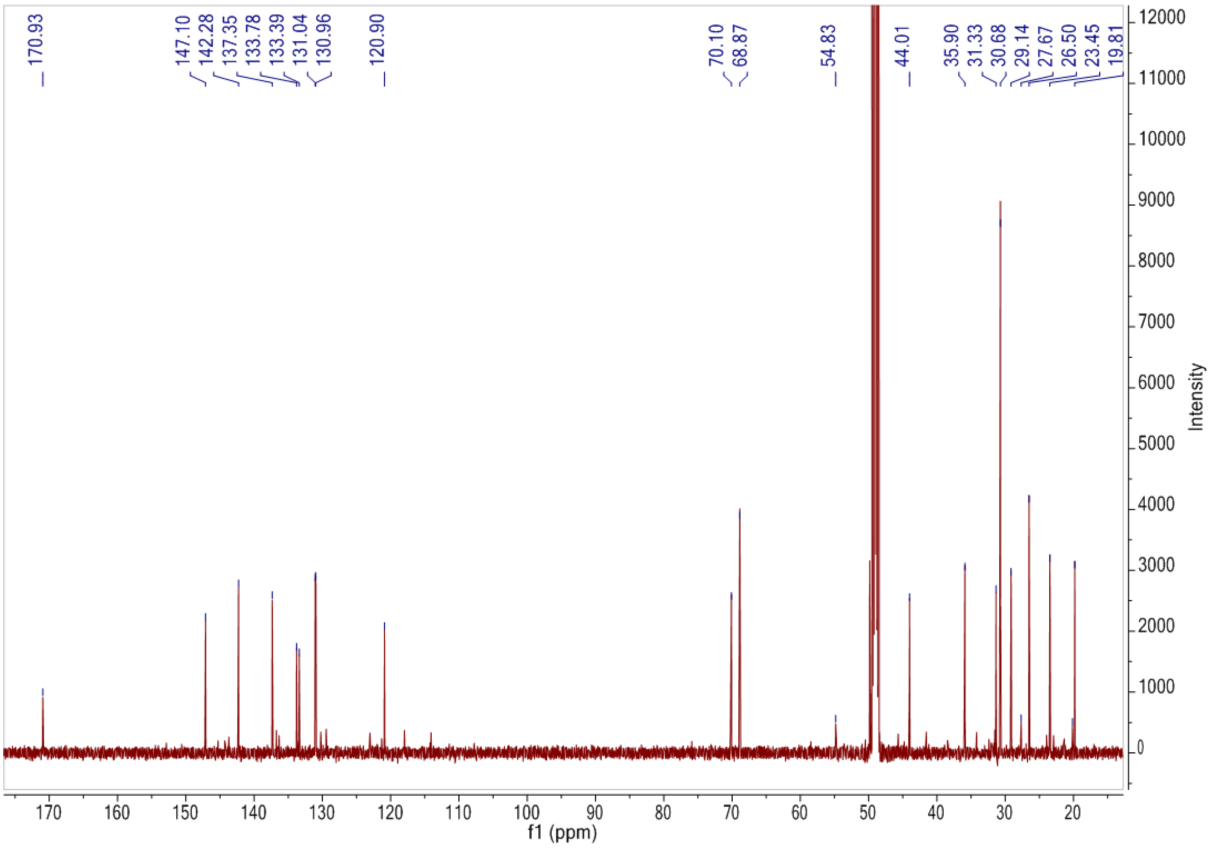

Figure S20: ^{13}C NMR spectrum of tanzawaic acid L (**4**) (CD_3OD).

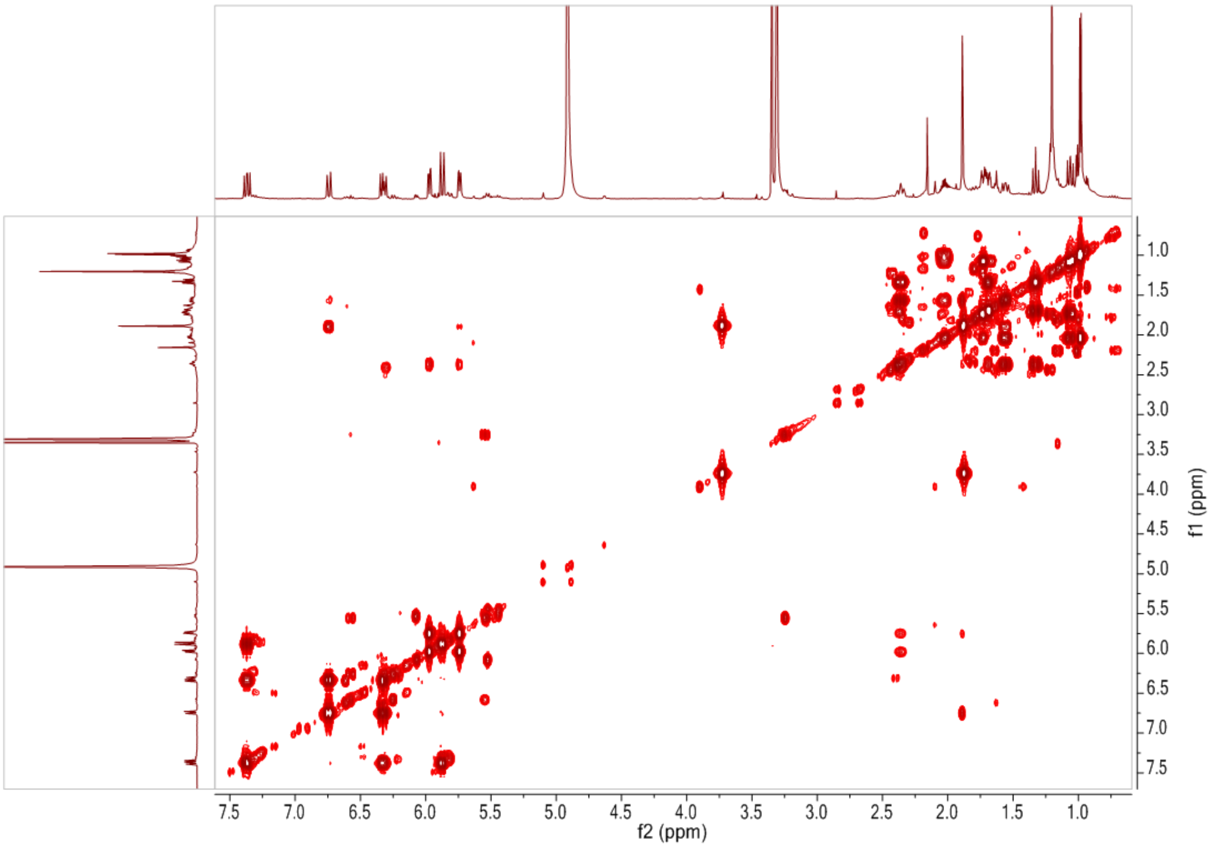

Figure S21: COSY spectrum of tanzawaic acid L (**4**) (CD_3OD).

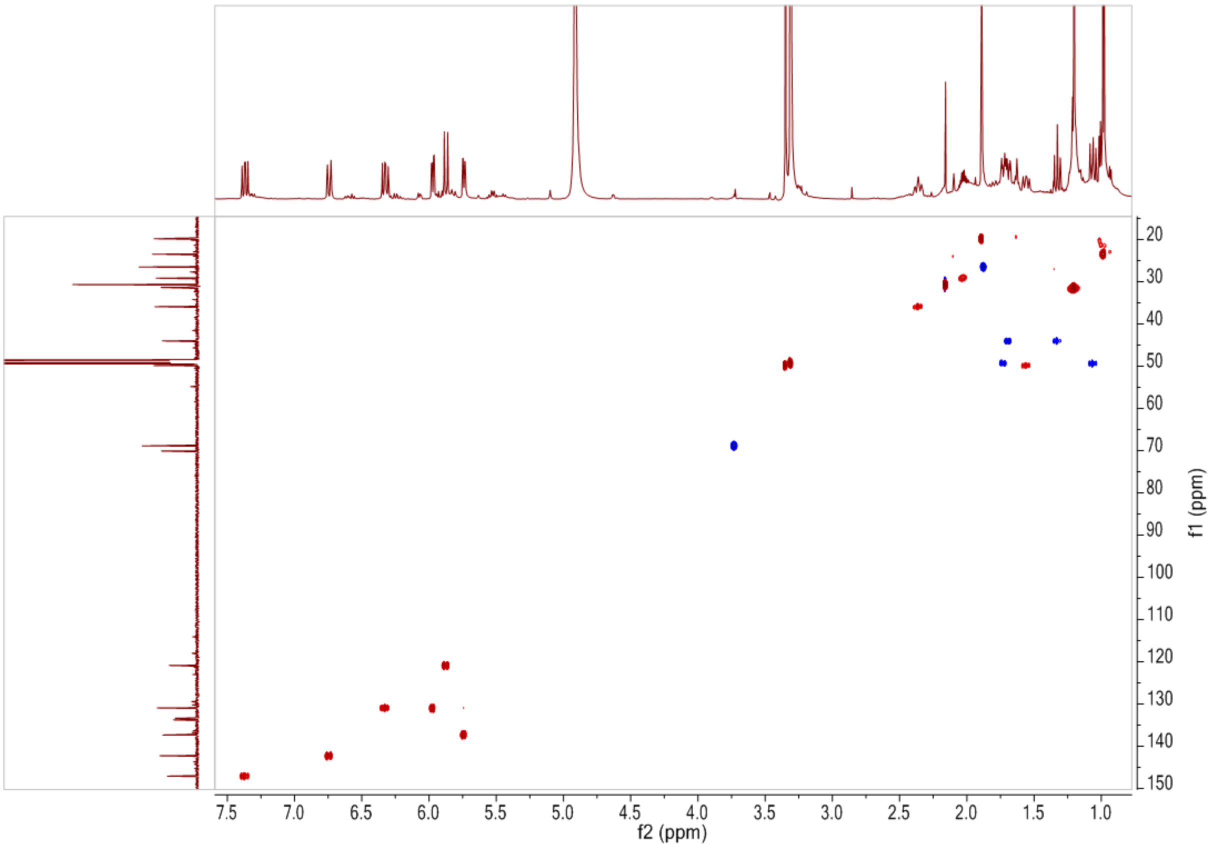

Figure S22: HSQC spectrum of tanzawaic acid L (**4**) (CD_3OD).

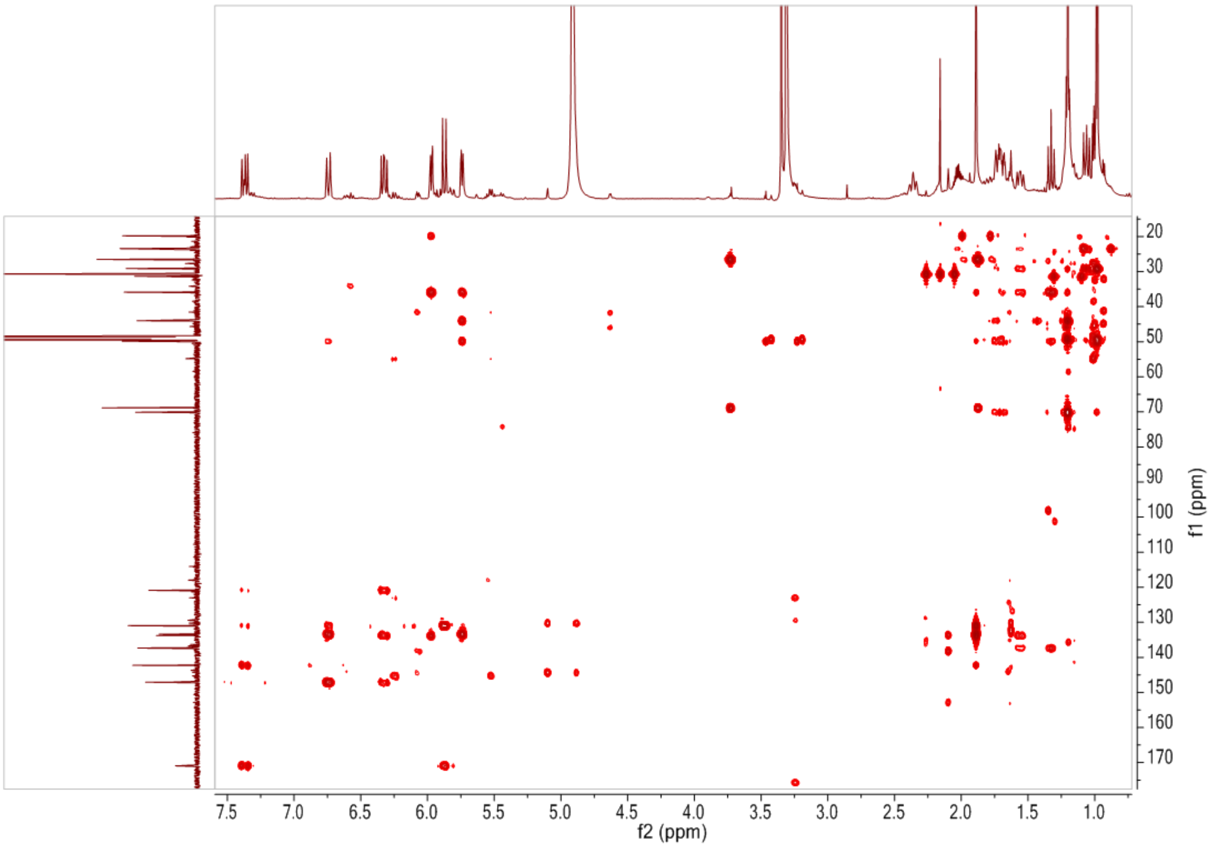

Figure S23: HMBC spectrum of tanzawaic acid L (**4**) (CD_3OD).

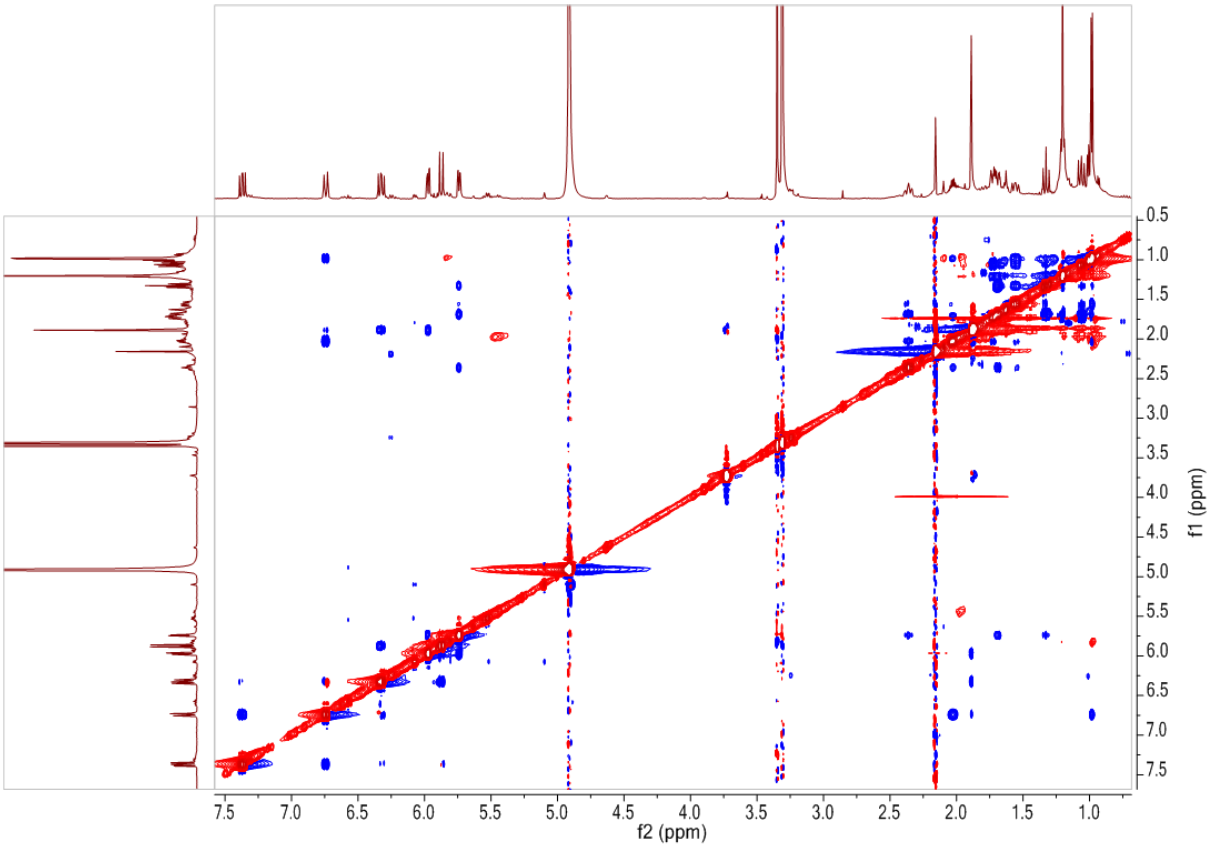

Figure S24: NOESY spectrum of tanzawaic acid L (**4**) (CD_3OD).

Crystal data for arohynapene A (**5**)

formula	$C_{18}H_{22}O_3$		
molecular weight	286.2 $gmol^{-1}$		
absorption	μ = 0.08 mm^{-1}		
crystal size	0.2 x 0.2 x 0.6 mm^3 colourless block		
space group	P $2_12_12_1$ (orthorhombic)		
lattice parameters	a =8.7599(5)Å		
(calculate from	b =13.3019(8)Å		
9956 reflections with	c =13.6416(8)Å		
2.3° < θ < 27.4°)	V = 1586.0(2)$Å^3$	z = 4	F(000) = 616
temperature	-100°C		
density	d_{xray} = 1.197 gcm^{-3}		

Data Collection

diffractometer	Smart CCD
radiation	Mo-K_α graphit monochromator
scan type	φ,ω-scans
scan – width	0.5°
scan range	2° ≤ θ < 28°
	-11 ≤ h ≤ 11 -17 ≤ k ≤ 17 -17 ≤ l ≤ 17
number of reflections:	
measured	28907
unique	3779 (R_{int} = 0.0291)
observed	3518 (|F|/σ(F) > 4.0)

Data correction, structure solution and refinement

Corrections	Lorentz and polarisation correction.
Structure solution	Program: SIR-97 (Direct methods)
Refinement	Program: SHELXL-97 (full matrix). 195 refined parameters, weighting scheme: $w=1/[\sigma^2(F_o^2) + (0.0507*P)^2+0.23*P]$ with $(Max(F_o^2,0)+2*F_c^2)/3$. H-atoms at calculated positions and refined with isotropic displacement parameters, non H-atoms refined anisotropically.
R-values	wR2 = 0.087 (R1 = 0.0324 for observed reflections, 0.0365 for all reflections)
goodness of fit	S = 1.033
maximum deviation	
of parameters	0.001 * e.s.d

maximum peak height in diff. Fourier synthesis	0.2, -0.18 eÅ^{-3}

final coordinates and equivalent displacement parameters (Å^2)

$$U_{äq} = (1/3)*\sum\sum_{ij} a_i^* a_j^* \mathbf{a}_i \mathbf{a}_j$$

Atom	X	Y	Z	U_{eq}
O1	0.5817(1)	1.16316(6)	0.57929(8)	0.0338(3)
O2	0.3349(1)	1.18042(7)	0.54487(9)	0.0414(3)
O3	0.5949(1)	0.36088(6)	0.57425(7)	0.0299(3)
C1	0.4466(2)	1.12719(9)	0.55665(9)	0.0264(3)
C2	0.4384(2)	1.01670(9)	0.54592(9)	0.0281(3)
C3	0.5571(1)	0.95547(8)	0.53585(9)	0.0241(3)
C4	0.5360(1)	0.84869(8)	0.51562(9)	0.0249(3)
C5	0.6438(1)	0.78568(9)	0.4857(1)	0.0260(3)
C6	0.6106(1)	0.68072(9)	0.45245(9)	0.0231(3)
C7	0.5684(1)	0.60376(8)	0.51880(8)	0.0217(3)
C8	0.5727(2)	0.62274(8)	0.62966(9)	0.0241(3)
C9	0.5121(2)	0.5342(1)	0.69151(9)	0.0301(4)
C10	0.3960(2)	0.46702(9)	0.63990(9)	0.0279(3)
C11	0.4682(1)	0.42578(8)	0.54645(9)	0.0243(3)
C12	0.5223(1)	0.51026(8)	0.48084(9)	0.0229(3)
C13	0.5245(2)	0.49394(9)	0.3796(1)	0.0290(4)
C14	0.5724(2)	0.5683(1)	0.3156(1)	0.0315(4)
C15	0.6150(2)	0.66257(9)	0.35063(9)	0.0277(4)
C16	0.6599(2)	0.7442(1)	0.2788(1)	0.0399(5)
C17	0.3371(2)	0.3835(1)	0.7073(1)	0.0383(4)
C18	0.7342(2)	0.6489(1)	0.6657(1)	0.0321(4)

anisotropic displacement parameters

Atom	U_{11}	U_{22}	U_{33}	U_{12}	U_{13}	U_{23}
O1	0.0312(5)	0.0137(4)	0.0566(6)	-0.0018(3)	-0.0037(5)	0.0030(4)
O2	0.0337(5)	0.0196(4)	0.0708(8)	0.0039(4)	-0.0125(5)	-0.0066(5)
O3	0.0299(5)	0.0166(4)	0.0433(5)	0.0031(3)	0.0028(4)	0.0037(4)
C1	0.0299(6)	0.0169(5)	0.0324(6)	0.0002(5)	-0.0010(5)	-0.0009(5)
C2	0.0285(6)	0.0174(5)	0.0383(7)	-0.0026(5)	-0.0003(5)	-0.0037(5)
C3	0.0268(6)	0.0168(5)	0.0287(6)	-0.0022(5)	0.0003(5)	0.0014(4)
C4	0.0263(6)	0.0158(5)	0.0326(6)	-0.0016(4)	-0.0017(5)	0.0001(4)
C5	0.0265(6)	0.0166(5)	0.0348(6)	-0.0009(4)	0.0008(5)	0.0034(5)
C6	0.0232(5)	0.0155(5)	0.0307(6)	0.0030(4)	0.0001(5)	-0.0001(4)
C7	0.0218(5)	0.0157(5)	0.0275(5)	0.0033(4)	0.0005(5)	-0.0010(4)
C8	0.0309(6)	0.0156(5)	0.0257(5)	0.0014(5)	0.0000(5)	-0.0026(4)
C9	0.0418(8)	0.0215(6)	0.0270(6)	-0.0008(5)	-0.0009(5)	0.0013(5)
C10	0.0312(6)	0.0197(5)	0.0328(6)	0.0016(5)	0.0036(5)	0.0029(5)
C11	0.0256(6)	0.0149(5)	0.0324(6)	-0.0001(4)	-0.0008(5)	-0.0002(4)
C12	0.0241(6)	0.0165(5)	0.0281(6)	0.0018(4)	-0.0005(5)	-0.0009(4)
C13	0.0350(7)	0.0206(6)	0.0314(6)	0.0010(5)	-0.0031(5)	-0.0052(5)
C14	0.0387(7)	0.0299(6)	0.0259(6)	0.0044(6)	-0.0009(5)	-0.0030(5)

C15	0.0302(6)	0.0238(6)	0.0290(6)	0.0041(5)	0.0015(5)	0.0042(5)
C16	0.0520(9)	0.0332(8)	0.0346(7)	-0.0016(7)	0.0050(7)	0.0085(6)
C17	0.0460(9)	0.0292(7)	0.0398(8)	-0.0065(6)	0.0090(7)	0.0043(6)
C18	0.0363(7)	0.0292(7)	0.0308(7)	-0.0020(5)	-0.0053(5)	-0.0019(6)

final coordinates and isotropic displacement parameters ($Å^2$) for H- atoms

Atom	X	Y	Z	U_{iso}
H1O	0.57874	1.22627	0.57846	0.0507
H3O	0.66110	0.36132	0.52969	0.0449
H2	0.33979	0.98697	0.54627	0.0337
H3	0.65758	0.98167	0.54192	0.0289
H4	0.43639	0.82195	0.52464	0.0299
H5	0.74669	0.80822	0.48537	0.0312
H8	0.50599	0.68210	0.64332	0.0289
H9A	0.59978	0.49226	0.71200	0.0361
H9B	0.46441	0.56162	0.75164	0.0361
H10	0.30703	0.50975	0.62061	0.0335
H11	0.39059	0.38497	0.51034	0.0292
H13	0.49266	0.43078	0.35430	0.0348
H14	0.57614	0.55496	0.24721	0.0378
H16A	0.64860	0.71903	0.21167	0.0599
H16B	0.76644	0.76351	0.29013	0.0599
H16C	0.59382	0.80286	0.28811	0.0599
H17A	0.42282	0.34143	0.72855	0.0575
H17B	0.26301	0.34207	0.67171	0.0575
H17C	0.28780	0.41346	0.76478	0.0575
H18C	0.80499	0.59524	0.64687	0.0482
H18B	0.73342	0.65575	0.73725	0.0482
H18A	0.76707	0.71241	0.63605	0.0482

Crystal data for tanzawaic acid E

formula	$C_{18}H_{26}O_3$
molecular weight	290.40 $gmol^{-1}$
absorption	μ = 0.61 mm^{-1}
crystal size	0.16 x 0.254 x 0.44 mm^3 colourless block
space group	P 2_1 2_1 2_1 (orthorhombic)
lattice parameters	a =8.8767(4)Å
(calculate from	b =12.3684(7)Å
16443 reflections with	c =15.3494(9)Å
2.88° < θ < 68.05°)	V = 1685.22(15)$Å^3$ z = 4 F(000) = 632
temperature	-80°C
density	d_{xray} = 1.145gcm^{-3}

data collection

diffractometer	STOE IPDS2T
radiation	Cu-K_α
Scan – type	ω scans
Scan – width	1°
scan range	$2° \leq \theta < 68°$
	$-13 \leq h \leq 14$ $-9 \leq k \leq 10$ $-17 \leq l \leq 18$
number of reflections:	
measured	9900
unique	2890 (R_{int} = 0.0460)
observed	2699 ($\|F\|/\sigma(F) > 4.0$)

Data correction, structure solution and refinement

corrections	Lorentz and polarisation correction.
Structure solution	Program: SIR-97 (Direct methods)
refinement	Program: SHELXL-97 (full matrix). 193 refined parameters, weighting scheme:
	$w=1/[\sigma^2(F_o^2) + (0.0979*P)^2+0.14*P]$ with $(Max(F_o^2,0)+2*F_o^2)/3$. H-atoms at calculated positions and refined with isotropic displacement parameters, non H-atoms refined anisotropically.
R-values	wR2 = 0.1305 (R1 = 0.0480 for observed reflections, 0.0502 for all reflections)
goodness of fit	S = 1.080
Flack parameter	x = 0.2(2)
maximum deviation	
of parameters	0.001 * e.s.d
maximum peak height in diff. Fourier synthesis	0.19, -0.27 $eÅ^{-3}$

final coordinates and equivalent displacement parameters (Å^2)

$U_{äq} = (1/3)*\sum\sum_{ij} a_i^* a_j^* \mathbf{a}_i \mathbf{a}_j$

Atom	X	Y	Z	U_{eq}
C1	0.5484(2)	0.3368(2)	0.1694(1)	0.0390(5)
C2	0.7024(2)	0.3650(1)	0.2110(1)	0.0366(5)
C3	0.7032(2)	0.4569(2)	0.2792(1)	0.0397(6)
C4	0.8632(2)	0.4697(2)	0.3174(1)	0.0412(6)
C5	0.9359(2)	0.3678(2)	0.3541(1)	0.0392(6)
C6	0.9256(2)	0.2776(2)	0.2869(1)	0.0386(5)
C7	0.7651(2)	0.2612(2)	0.2532(1)	0.0373(5)
C8	0.7581(2)	0.1656(2)	0.1931(1)	0.0455(6)
C9	0.6722(3)	0.1616(2)	0.1226(1)	0.0468(6)
C10	0.5680(2)	0.2511(2)	0.0954(1)	0.0429(6)
C11	0.4564(2)	0.4282(2)	0.1332(1)	0.0397(6)
C12	0.3065(2)	0.4319(2)	0.1374(1)	0.0405(6)
C13	0.2137(2)	0.5104(2)	0.0923(1)	0.0394(6)
C14	0.0655(2)	0.4997(2)	0.0813(1)	0.0406(6)
C15	-0.0256(2)	0.5750(2)	0.0285(1)	0.0383(5)
O16	-0.1656(2)	0.5410(1)	0.01969(9)	0.0482(5)
O17	0.0206(2)	0.6583(1)	-0.0037(1)	0.0487(4)
C18	0.6555(3)	0.5689(2)	0.2454(2)	0.0507(7)
O19	0.8473(2)	0.3376(1)	0.42966(8)	0.0452(4)
C20	1.0969(3)	0.3885(2)	0.3822(2)	0.0499(7)
C21	0.6198(3)	0.2989(2)	0.0080(1)	0.0516(7)

anisotropic displacement parameters

Atom	U_{11}	U_{22}	U_{33}	U_{12}	U_{13}	U_{23}
C1	0.0351(9)	0.0407(10)	0.0413(9)	-0.0019(8)	-0.0002(8)	0.0043(8)
C2	0.0338(10)	0.0377(9)	0.0383(9)	0.0005(7)	0.0039(7)	0.0002(7)
C3	0.040(1)	0.0394(10)	0.0398(9)	0.0050(8)	0.0011(8)	-0.0032(8)
C4	0.046(1)	0.0399(10)	0.0374(9)	-0.0017(8)	-0.0010(8)	-0.0050(8)
C5	0.0365(10)	0.0428(10)	0.0383(9)	0.0038(8)	0.0010(8)	-0.0014(8)
C6	0.0368(10)	0.0393(9)	0.0398(9)	0.0054(7)	0.0030(8)	0.0013(8)
C7	0.036(1)	0.0379(9)	0.0382(9)	0.0005(8)	-0.0002(7)	-0.0006(8)
C8	0.048(1)	0.0350(9)	0.054(1)	0.0053(9)	-0.0038(9)	-0.0050(9)
C9	0.053(1)	0.0376(10)	0.050(1)	0.0000(9)	-0.0046(9)	-0.0088(8)
C10	0.041(1)	0.044(1)	0.0430(10)	-0.0054(8)	-0.0047(8)	-0.0005(9)
C11	0.039(1)	0.044(1)	0.0367(9)	-0.0026(8)	-0.0010(8)	0.0007(8)
C12	0.038(1)	0.047(1)	0.0364(9)	0.0011(8)	0.0005(7)	-0.0016(8)
C13	0.039(1)	0.0459(10)	0.0339(9)	0.0021(8)	0.0015(7)	-0.0044(7)
C14	0.039(1)	0.045(1)	0.0379(9)	0.0043(8)	0.0013(8)	-0.0010(8)
C15	0.038(1)	0.0424(10)	0.0342(9)	0.0011(8)	-0.0001(7)	-0.0059(7)
O16	0.0370(8)	0.0507(8)	0.0570(8)	-0.0020(6)	-0.0082(6)	0.0120(7)
O17	0.0499(8)	0.0439(7)	0.0524(8)	-0.0076(6)	-0.0076(6)	0.0038(6)
C18	0.055(1)	0.040(1)	0.057(1)	0.0075(9)	-0.008(1)	-0.0078(9)
O19	0.0488(8)	0.0475(8)	0.0393(7)	0.0118(6)	0.0044(6)	0.0018(6)
C20	0.050(1)	0.050(1)	0.050(1)	-0.0014(9)	-0.0073(9)	-0.0013(9)

C21	0.063(1)	0.050(1)	0.042(1)	0.0006(10)	0.0024(10)	-0.0057(9)

final coordinates and isotropic displacement parameters (Å^2) for H- atoms

Atom	X	Y	Z	U_{iso}
H1	0.48634	0.30198	0.21592	0.0469
H2	0.77252	0.38622	0.16293	0.0439
H3	0.63363	0.43618	0.32760	0.0477
H4A	0.92958	0.49866	0.27112	0.0494
H4B	0.85896	0.52443	0.36439	0.0494
H6A	0.96170	0.20941	0.31336	0.0463
H6B	0.99253	0.29497	0.23722	0.0463
H7	0.70014	0.24437	0.30464	0.0448
H8	0.81884	0.10450	0.20632	0.0546
H9	0.67665	0.09854	0.08736	0.0562
H10	0.46689	0.21778	0.08538	0.0515
H11	0.50762	0.48653	0.10588	0.0477
H12	0.25706	0.37944	0.17245	0.0486
H13	0.26123	0.57297	0.06924	0.0473
H14	0.01605	0.44070	0.10885	0.0487
H16	-0.21444	0.58573	-0.01030	0.0723
H18A	0.68095	0.62398	0.28887	0.076
H18B	0.54657	0.56947	0.23501	0.076
H18C	0.70854	0.58453	0.19087	0.076
H19	0.88277	0.28084	0.45166	0.0678
H20A	1.09928	0.44899	0.42341	0.075
H20B	1.15821	0.40620	0.33111	0.075
H20C	1.13731	0.32355	0.41041	0.075
H21A	0.71031	0.34278	0.01708	0.0775
H21B	0.53948	0.34419	-0.01622	0.0775
H21C	0.64257	0.24004	-0.03273	0.0775
